# Supplementary material for: Integrated Metabolomic, Molecular Networking, and Genome Mining Analyses Uncover Novel Angucyclines From Streptomyces sp. RO-S4 Strain Isolated From Bejaia Bay, Algeria
Source: Front Microbiol. 2022 Jun 23;13:906161. doi: 10.3389/fmicb.2022.906161 (PMC9260717; doi:10.3389/fmicb.2022.906161)
Supplement: Supplementary file 1 [file Data_Sheet_1.pdf]

Supporting Information for:

Integrated metabolomic, molecular networking, and genome mining analyses uncover novel angucyclines from *Streptomyces* sp. RO-S4 strain isolated from Bejaia Bay, Algeria

Rima Ouchene[1,2], Didier Stien[2*], Juliette Segret[2], Mouloud Kecha[1], Alice M. S. Rodrigues[2], Carole Veckerlé[2], Marcelino T. Suzuki[2*]

Table of content

Table S1. List and mass spectrometry annotation key of sugars constituting the angucycline ose moieties 3
Figure S1. MS spectrum of compound 1. 4
Figure S2. MS/MS spectrum of an ion at m/z 487.1600 [(1+H)$^+$]. 4
Figure S3. Compared experimental MS/MS spectrum of ion at m/z 487.1600 [(1+H)$^+$] with: (a) MS^2 spectrum of protonated fridamycin and (b) MS^2 spectrum of protonated aquayamycin. 5
Figure S4. MS spectrum of compound 2. 6
Figure S5. MS/MS spectrum of ion at m/z 469.1496 [(2+H)$^+$]. 6
Figure S6. MS spectrum of compound 3. 6
Figure S7. MS/MS spectrum of ion at m/z 529.1708 [(3+H)$^+$]. 6
Figure S8. MS spectrum of compound 4. 7
Figure S9. MS/MS spectrum of ion at m/z 557.2378 [(4+H)$^+$]. 7
Figure S10. MS spectrum of compound 5. 7
Figure S11. MS/MS spectrum of ion at m/z 669.2903 [(5+H)$^+$]. 7
Figure S12. MS spectrum of compound 6a. 8
Figure S13. MS/MS spectrum of ion at m/z 597.1973 [(6a+H)$^+$]. 8
Figure S14. MS spectrum of compound 6b. 8
Figure S15. MS/MS spectrum of ion at m/z 597.1967 [(6b+H)$^+$]. 8
Figure S16. MS spectrum of compound 7a. 9
Figure S17. MS/MS spectrum of ion at m/z 711.2650 [(7a+H)$^+$]. 9
Figure S18. MS spectrum of compound 7b. 9
Figure S19. MS/MS spectrum of ion at m/z 711.2656 [(7b+H)$^+$]. 9
Figure S20. MS spectrum of compound 8. 10
Figure S21. MS/MS spectrum of ion at m/z 545.1808 [(8+H)$^+$]. 10
Figure S22. MS spectrum of compound 9. 10
Figure S23. MS/MS spectrum of ion at m/z 667.2751 [(9+H)$^+$]. 10
Figure S24. MS spectrum of compound 10. 11
Figure S25. MS/MS spectrum of ion at m/z 781.3436 [(10+H)$^+$]. 11
Figure S26. MS spectrum of compound 11a. 11
Figure S27. MS/MS spectrum of ion at m/z 675.2438 [(11a+H)$^+$]. 11
Figure S28. MS spectrum of compound 11b. 12
Figure S29. MS/MS spectrum of ion at m/z 675.2436 [(11b+H)$^+$]. 12
Figure S30. MS spectrum of compound 12. 12
Figure S31. MS/MS spectrum of ion at m/z 677.2597 [(12+H)$^+$]. 12
Figure S32. MS spectrum of compound 13. 13
Figure S33. MS/MS spectrum of ion at m/z 763.3328 [(13+H)$^+$]. 13
Figure S34. MS spectrum of compound 14. 13
Figure S35. MS/MS spectrum of ion at m/z 917.3601 [(14+H)$^+$]. 13

Figure S36. MS spectrum of compound 15a. 14
Figure S37. MS/MS spectrum of ion at m/z 819.2863 [(15a+H)+]. 14
Figure S38. MS spectrum of compound 15b. 14
Figure S39. MS/MS spectrum of ion at m/z 819.2869 [$(15b+H)^+$]. 14
Figure S40. MS spectrum of compound 16. 15
Figure S41. MS/MS spectrum of ion at m/z 901.3653 [$(16+H)^+$]. 15
Figure S42. MS spectrum of compound 17. 15
Figure S43. MS/MS spectrum of ion at m/z 952.3960 [$(17+NH_4)^+$]. 15
Figure S44. MS/MS spectrum of ion at m/z 957.3506 [$(17+Na)^+$]. 16
Figure S45. MS spectrum of compound 18a. 16
Figure S46. MS/MS spectrum of ion at m/z 599.2142 [$(18a+H)^+$]. 16
Figure S47. MS spectrum of compound 18b. 16
Figure S48. MS/MS spectrum of ion at m/z 599.2125 [$(18b+H)^+$]. 17
Figure S49. MS spectrum of compound 19a. 17
Figure S50. MS/MS spectrum of ion at m/z 601.2284 [$(19a+H)^+$]. 17
Figure S51. MS spectrum of compound 19b. 17
Figure S52. MS/MS spectrum of ion at m/z 601.2288 [$(19b+H)^+$]. 18
Figure S53. MS spectrum of compound 20. 18
Figure S54. MS/MS spectrum of ion at m/z 583.2170 [$(20+H)^+$]. 18
Figure S55. MS spectrum of compound 21. 18
Figure S56. MS/MS spectrum of ion at m/z 697.2861 [$(21+H)^+$]. 19
Figure S57. MS spectrum of compound 22. 19
Figure S58. MS/MS spectrum of ion at m/z 585.2333 [$(22+H)^+$]. 19
Figure S59. MS spectrum of compound 23. 19
Figure S60. MS/MS spectrum of ion at m/z 713.2782 [$(23+H)^+$]. 20
Figure S61. MS spectrum of compound 24. 20
Figure S62. MS/MS spectrum of ion at m/z 672.2655 [$(24+H)^+$] 20
Figure S63. MS spectrum of compound 25. 20
Figure S64. MS/MS spectrum of ion at m/z 743.3031 [$(25+H)^+$]. 21
Figure S65. Fragmentation scheme for compound 25 from $[M+H]^+$ to the aglycone ion. 21
Figure S66. MS spectrum of compound 26. 21
Figure S67. MS/MS spectrum of ion at m/z 813.3816 [$(26+H)^+$]. 21
Figure S68. MS spectrum of compound 27. 22
Figure S69. MS/MS spectrum of ion at m/z 857.3709 [$(27+H)^+$]. 22
Figure S70. MS spectrum of compound 28. 22
Figure S71. MS/MS spectrum of ion at m/z 709.2490 [$(28+H)^+$]. 22
Figure S72. MS spectrum of compound 29. 23
Figure S73. MS/MS spectrum of ion at m/z 595.1808 [$(29+H)^+$]. 23
Figure S74. MS spectrum of compound 30. 23
Figure S75. MS/MS spectrum of ion at m/z 705.2182 [$(30+H)^+$]. 23
Table S2. Summary of antiSMASH 6.0 secondary metabolites predicted from the RO-S4 strain 24
Table S3. Deduced functions of the ORFs in the ROS4_2 cluster ‘Streptomyces sp. RO-S4 strain and their BLASTs. We have considered only the sequences that showed a homology >50% 25
Figure S76. $tRNA_{ala}$ predicted by Aragorn in the complementary strand of the LRR80_00492 ORF region. 33
Figure S77. RNA-fold predicted RNA secondary structure of the region predicted by Aragorn to code (in the complementary strand) a tRNAala in the LRR80_00492 ORF region gene. 34
Figure S78. Phylogenetic reconstruction (Neighbor Joining) of FAD dependent oxidases as described in the text. Where: 36
Figure S79. Phylogenetic reconstruction (Maximum Likelihood) of FAD dependent oxidases as described in the text. 38

Table S1. List and mass spectrometry annotation key of sugars constituting the angucycline ose moieties

Name	Neutral loss 1	Neutral loss 2	Oxonium cation
**Aculose**	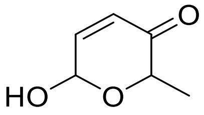 Exact Mass: 128.0473	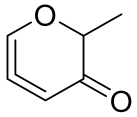 Exact Mass: 110.0368	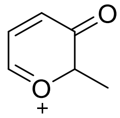 Exact Mass: 111.0441
**Oxidized aculose ($C_6H_8O_4$)**	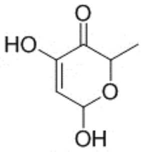 Chemical Formula: $C_6H_8O_4$ Exact Mass: 144.0423	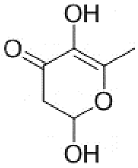 Chemical Formula: $C_6H_8O_4$ Exact Mass: 144.0423	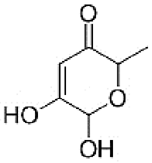 Chemical Formula: $C_6H_8O_4$ Exact Mass: 144.0423
**Rhodinose or amicetose (diastereoisomers)**	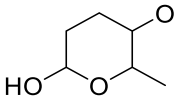 Exact Mass: 132.0786	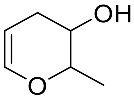 Exact Mass: 114.0681	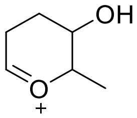 Exact Mass: 115.0754
**Cinerulose A**	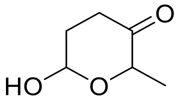 Exact Mass: 130.0630	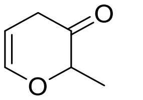 Exact Mass: 112.0524	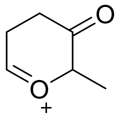 Exact Mass: 113.0597
**Cinerulose B**	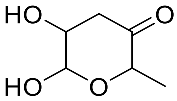 Exact Mass: 146.0579	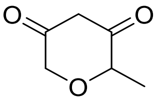 Exact Mass: 128.0473	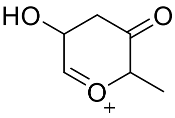 Exact Mass: 129.0546
**Olivose**	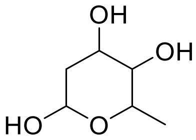 Exact Mass: 148.0736	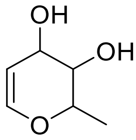 Exact Mass: 130.0630	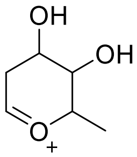 Exact Mass: 131.0703
**Kerriose**	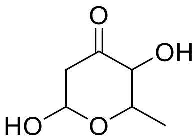 Exact Mass: 146.0579	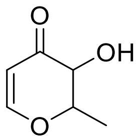 Exact Mass: 128.0473	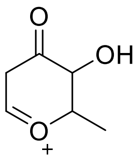 Exact Mass: 129.0546

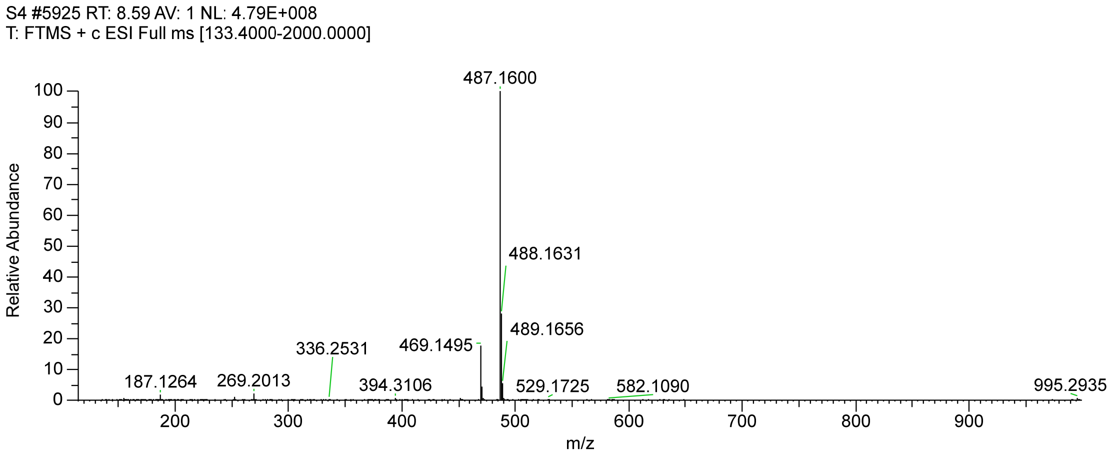

Figure S1. MS spectrum of compound **1.**

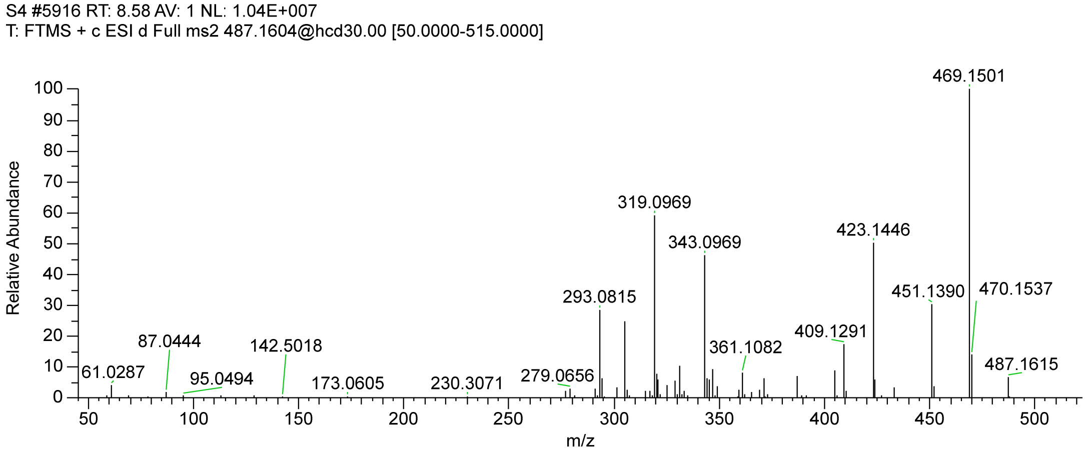

Figure S2. MS/MS spectrum of an ion at *m/z* 487.1600 [(**1**+H)$^{+}$].

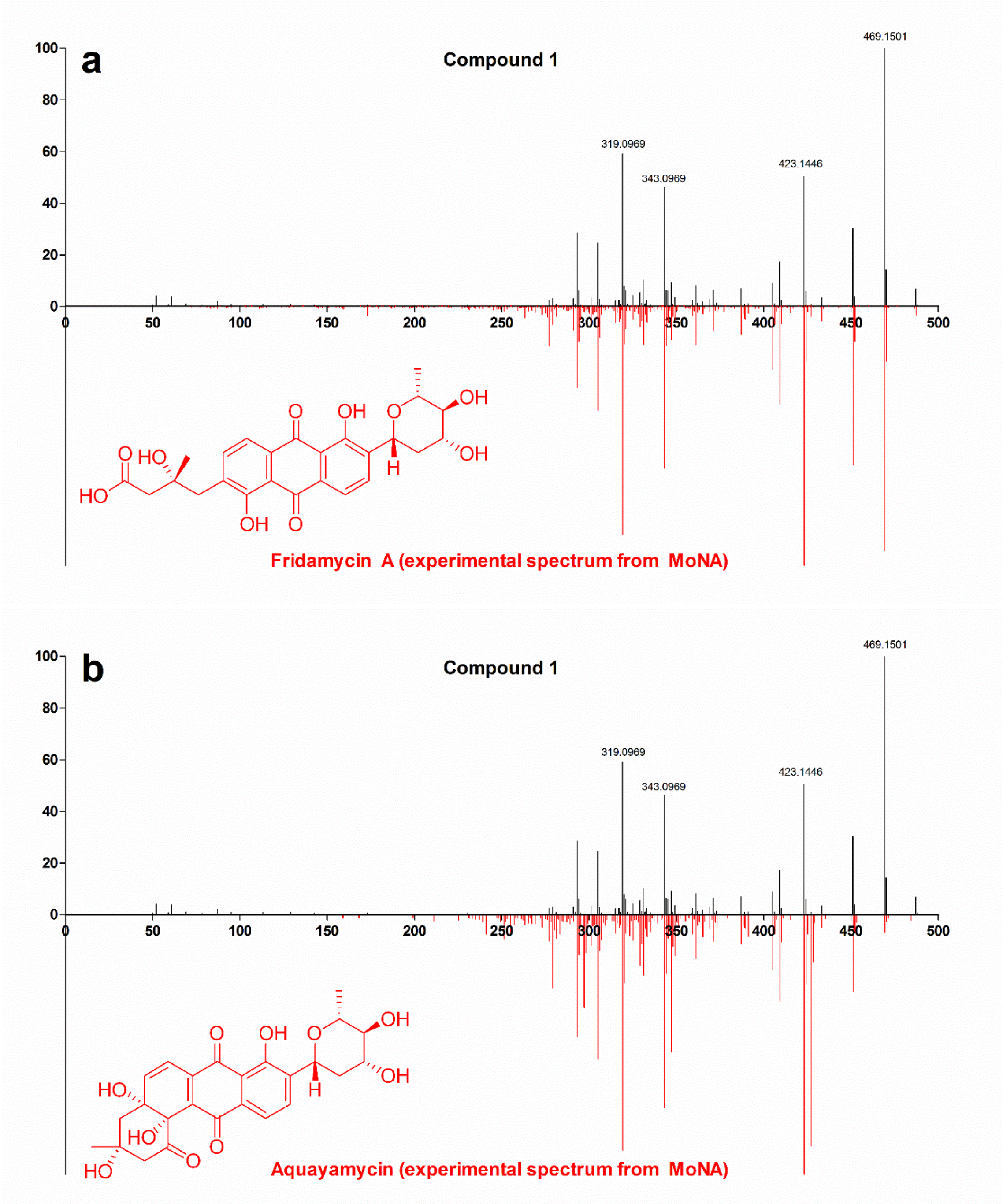

Figure S3. Compared experimental MS/MS spectrum of ion at *m/z* 487.1600 [(**1**+H)$^+$] with: (a) MS^2 spectrum of protonated fridamycin and (b) MS^2 spectrum of protonated aquayamycin.

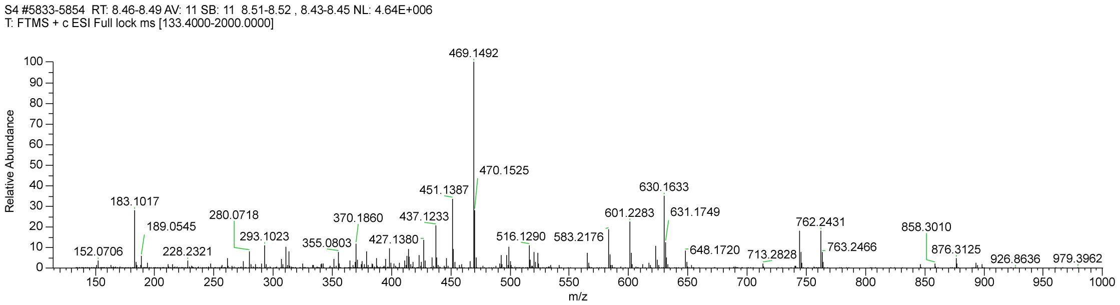

Figure S4. MS spectrum of compound **2**.

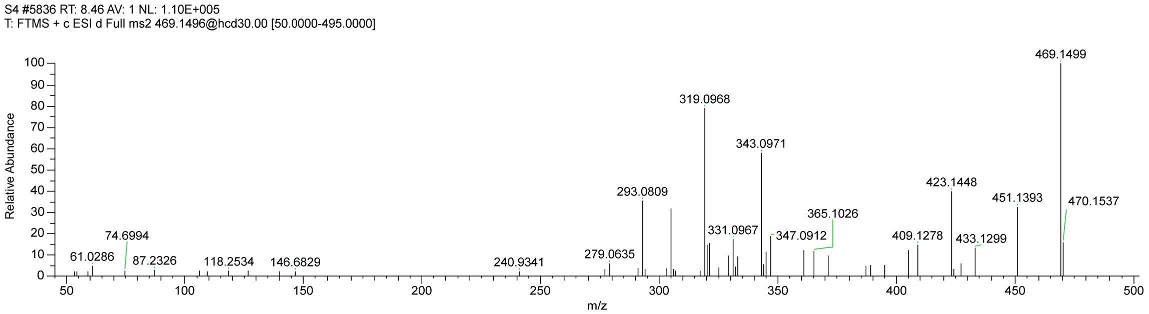

Figure S5. MS/MS spectrum of ion at *m/z* 469.1496 [(**2**+H)$^+$].

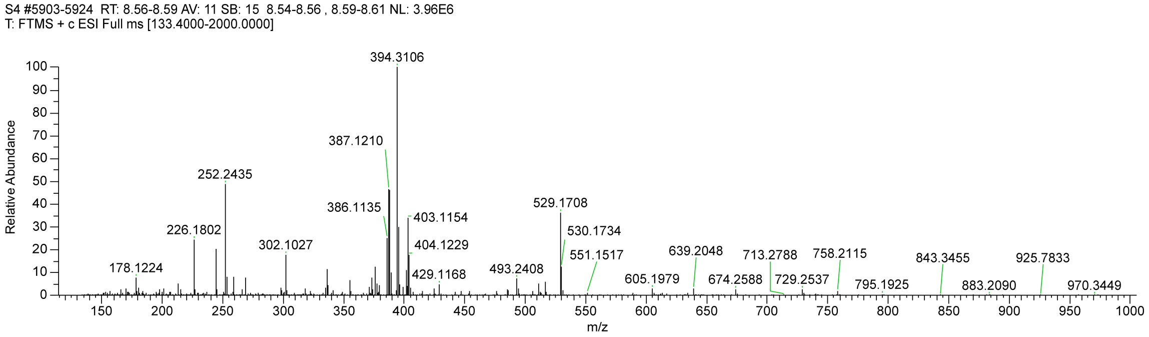

Figure S6. MS spectrum of compound **3**.

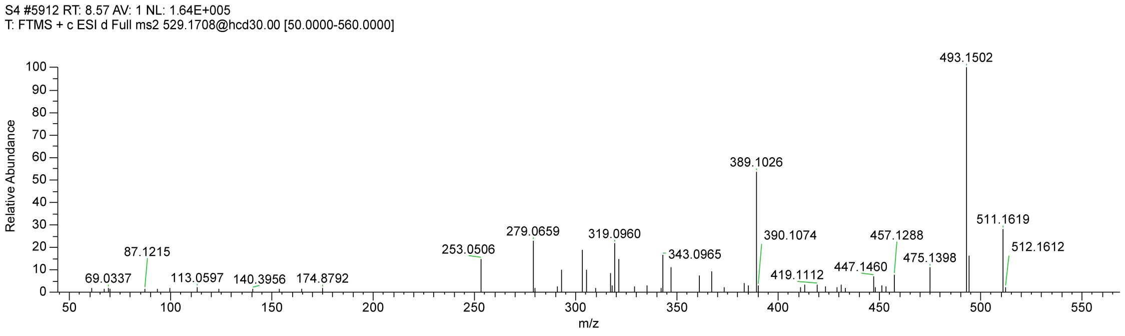

Figure S7. MS/MS spectrum of ion at *m/z* 529.1708 [(**3**+H)$^+$].

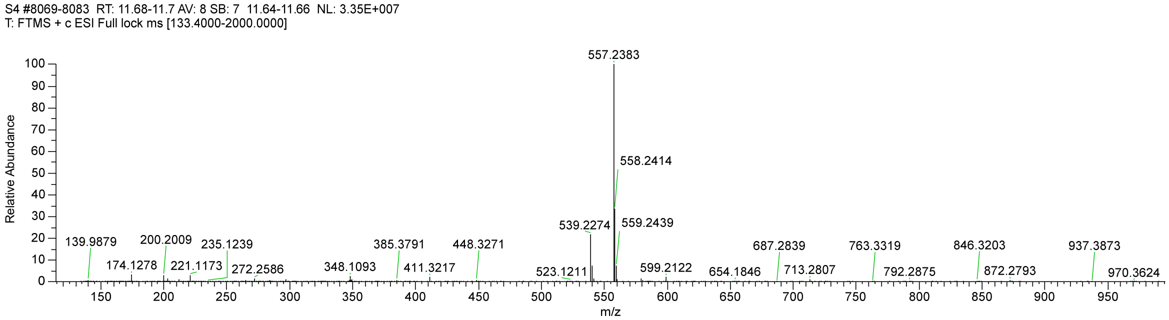

Figure S8. MS spectrum of compound **4**.

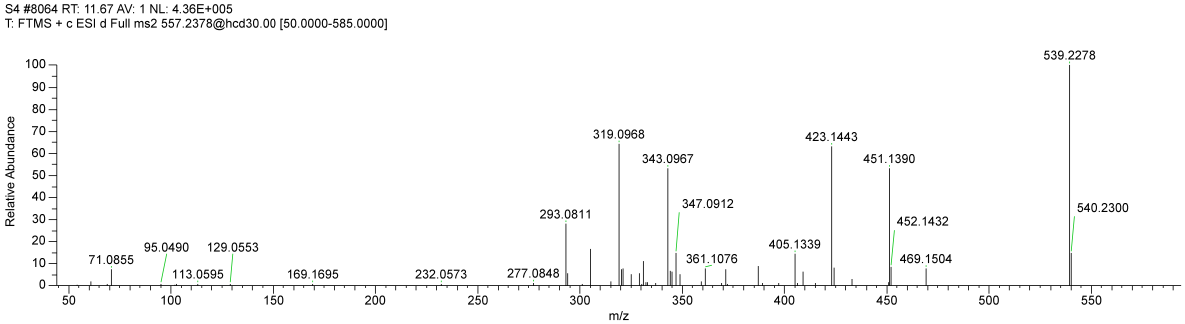

Figure S9. MS/MS spectrum of ion at *m/z* 557.2378 [(**4**+H)$^+$].

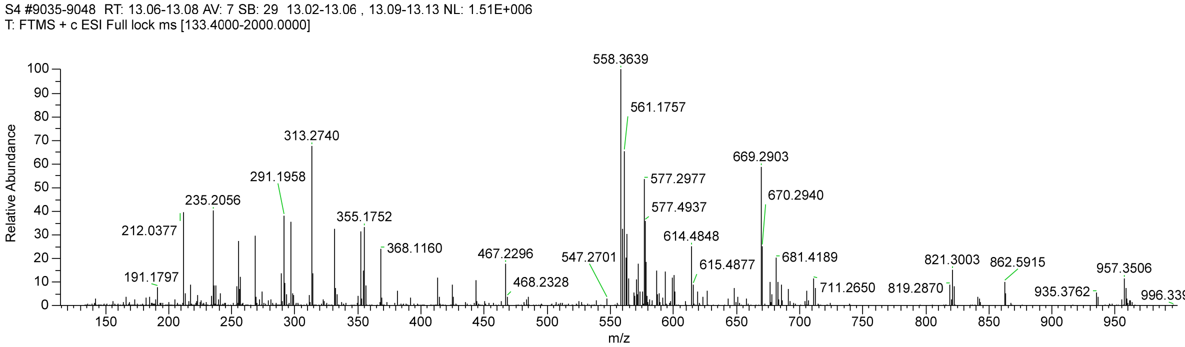

Figure S10. MS spectrum of compound **5**.

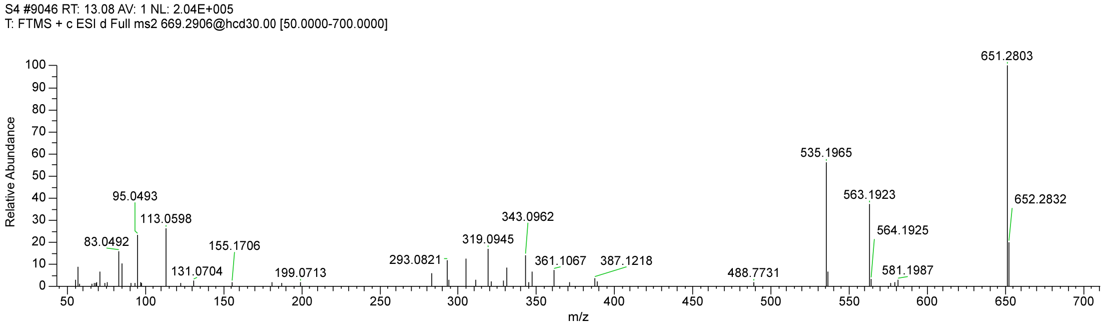

Figure S11. MS/MS spectrum of ion at *m/z* 669.2903 [(**5**+H)$^+$].

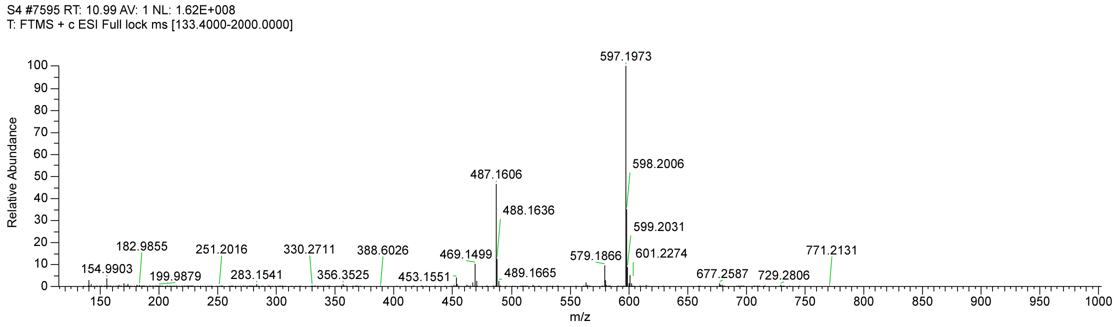

Figure S12. MS spectrum of compound **6a**.

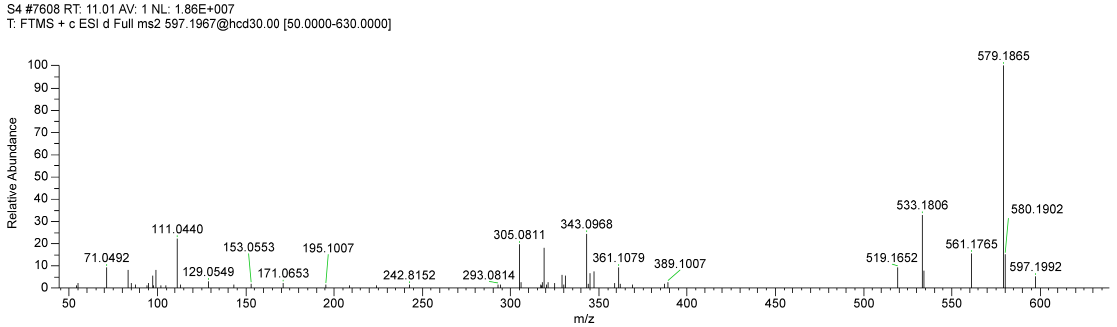

Figure S13. MS/MS spectrum of ion at *m/z* 597.1973 [(**6a**+H)$^+$].

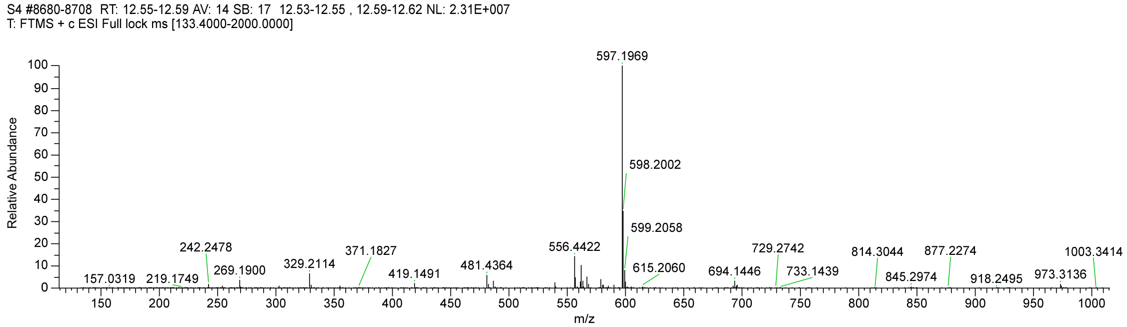

Figure S14. MS spectrum of compound **6b**.

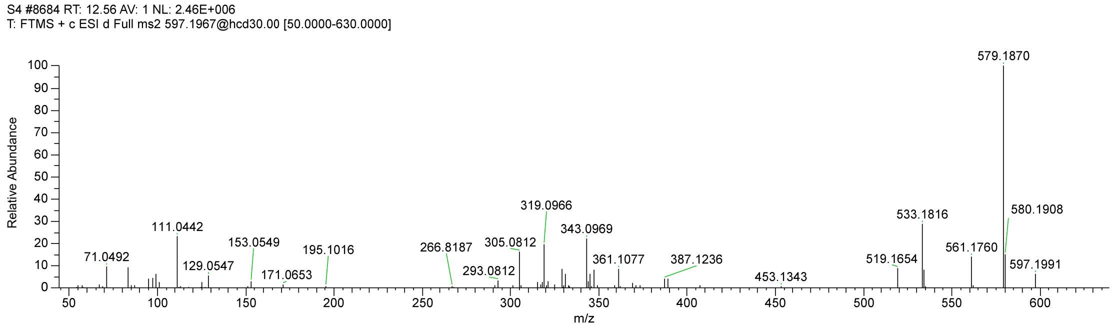

Figure S15. MS/MS spectrum of ion at *m/z* 597.1967 [(**6b**+H)$^+$].

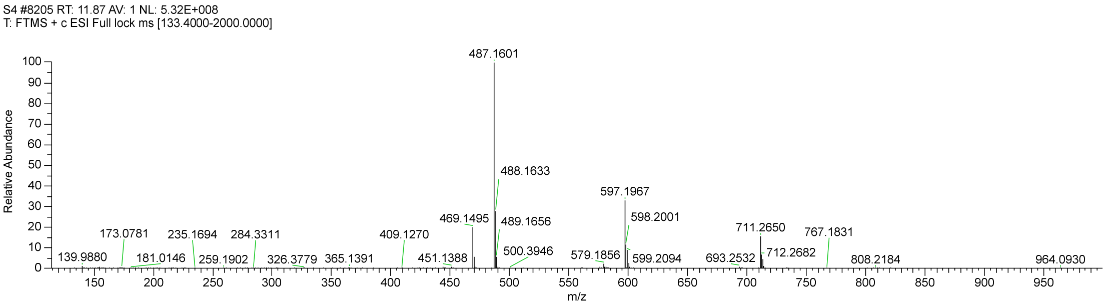

Figure S16. MS spectrum of compound **7a**.

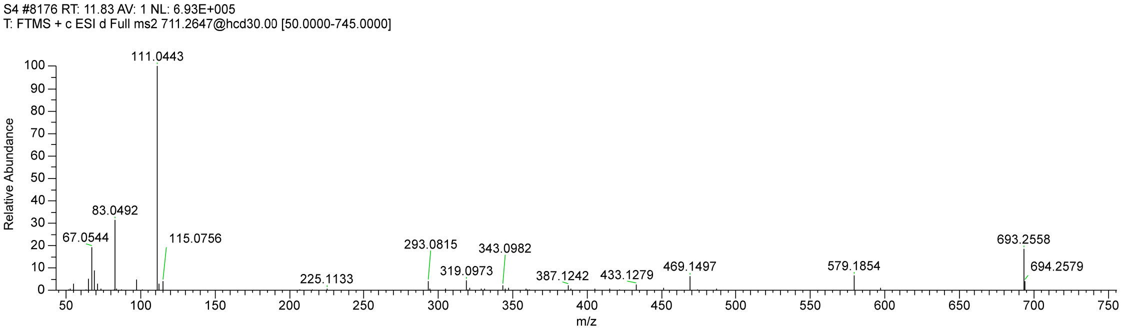

Figure S17. MS/MS spectrum of ion at *m/z* 711.2650 [(**7a**+H)$^+$].

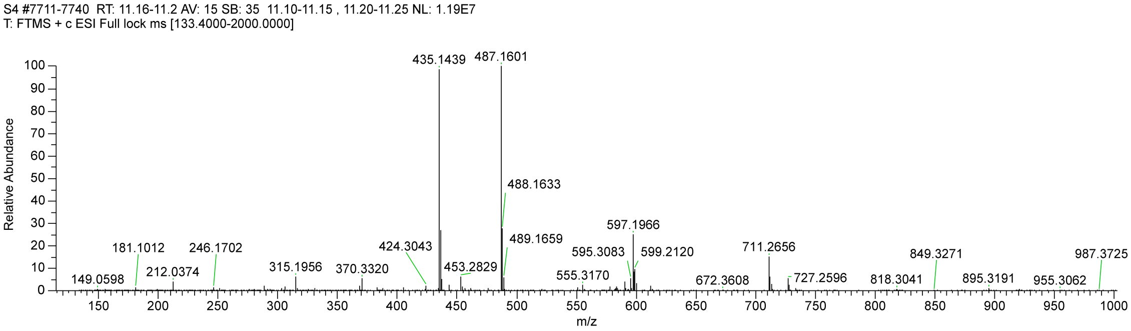

Figure S18. MS spectrum of compound **7b**.

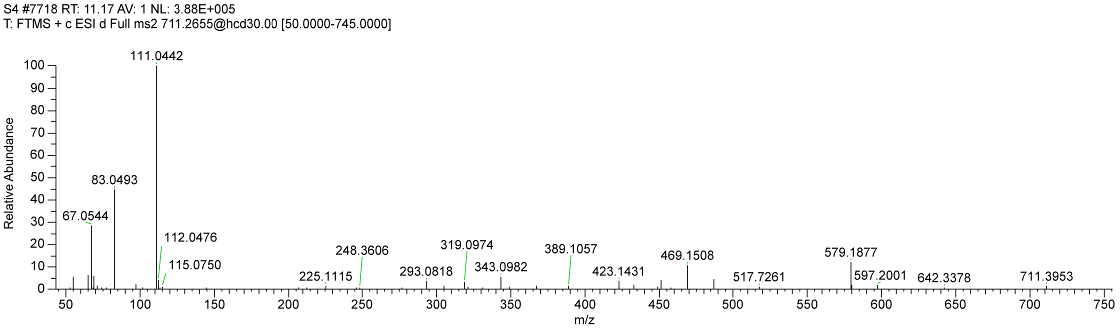

Figure S19. MS/MS spectrum of ion at *m/z* 711.2656 [(**7b**+H)$^+$].

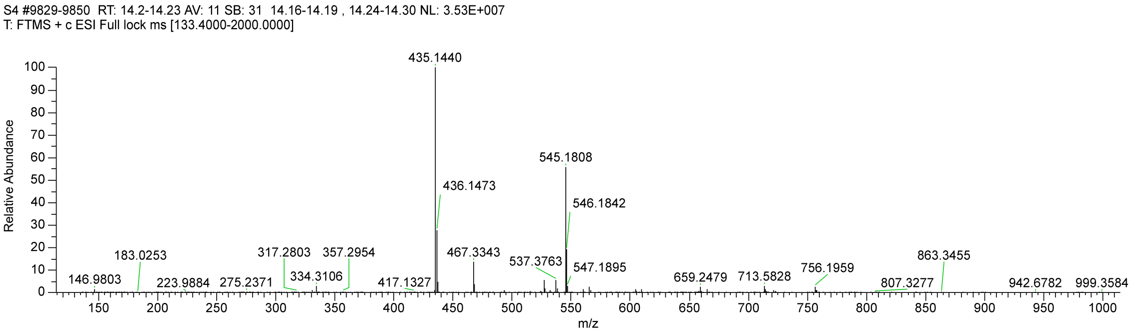

Figure S20. MS spectrum of compound **8**.

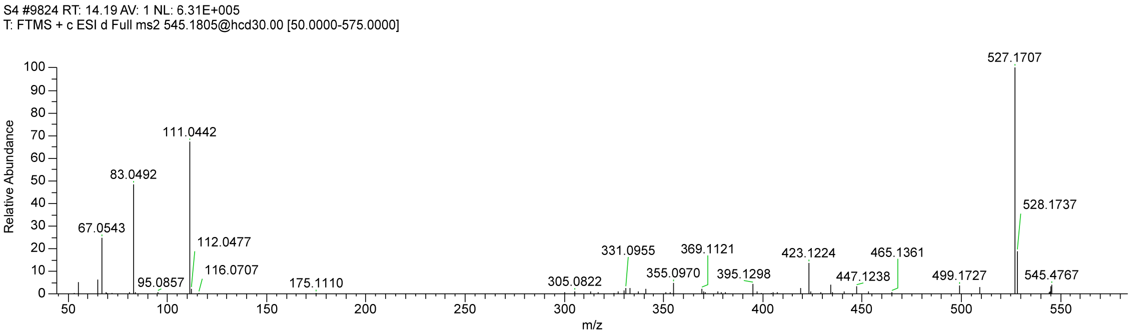

Figure S21. MS/MS spectrum of ion at *m/z* 545.1808 [(**8**+H)$^{+}$].

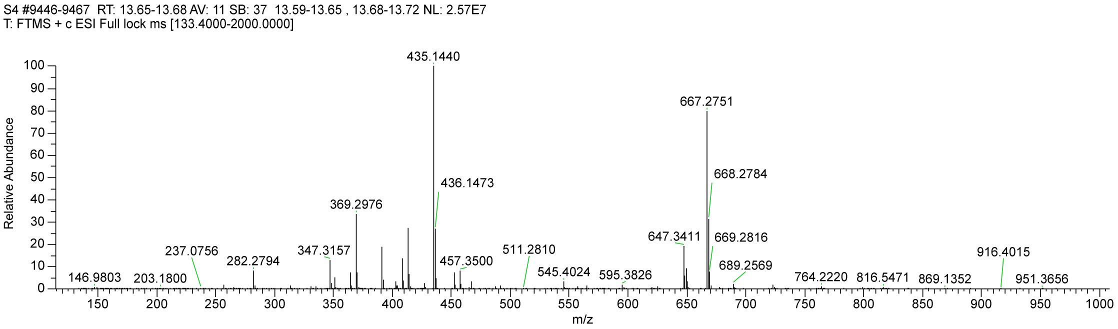

Figure S22. MS spectrum of compound **9**.

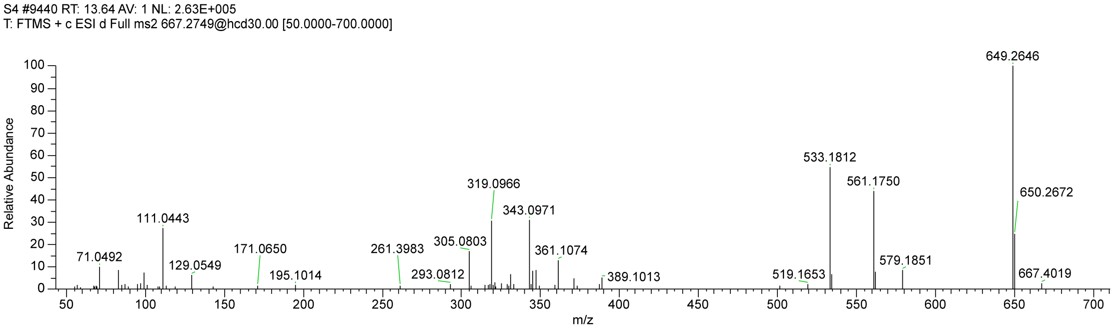

Figure S23. MS/MS spectrum of ion at *m/z* 667.2751 [(**9**+H)$^{+}$].

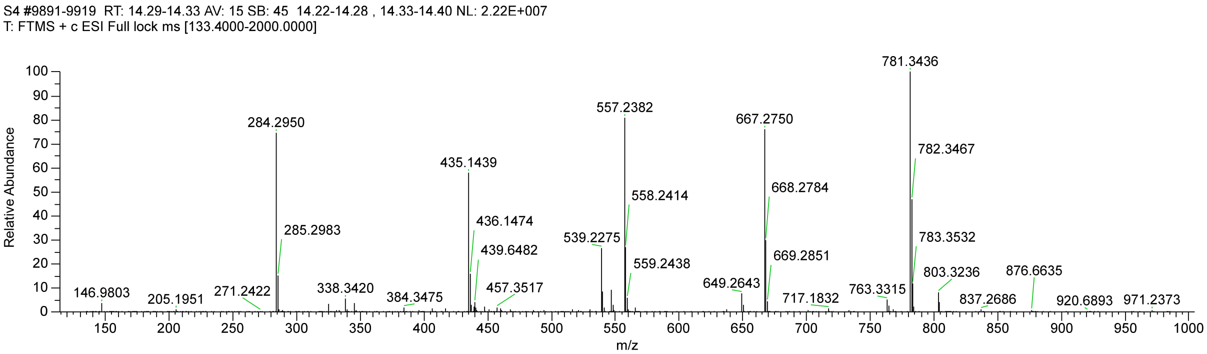

Figure S24. MS spectrum of compound **10**.

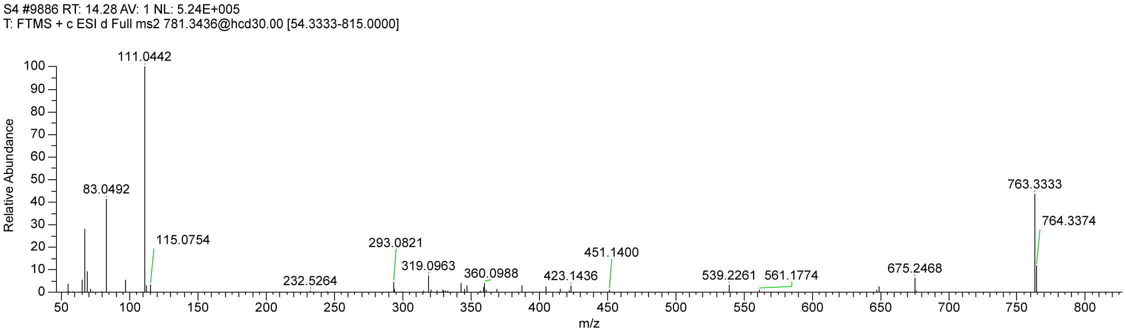

Figure S25. MS/MS spectrum of ion at *m/z* 781.3436 [(**10**+H)$^+$].

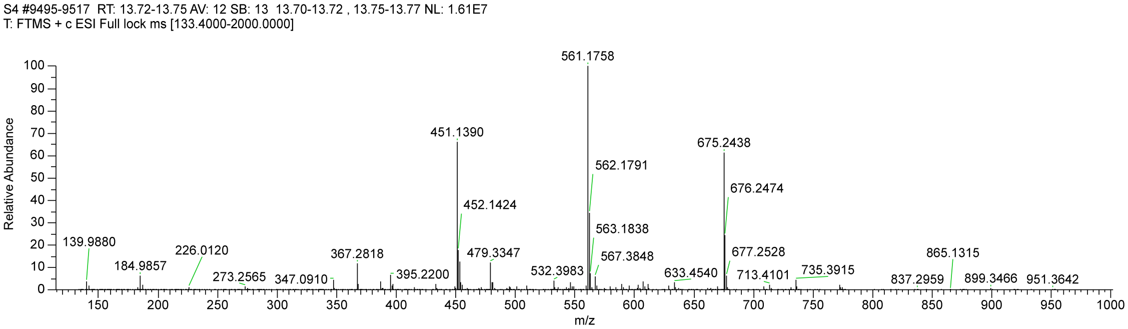

Figure S26. MS spectrum of compound **11a**.

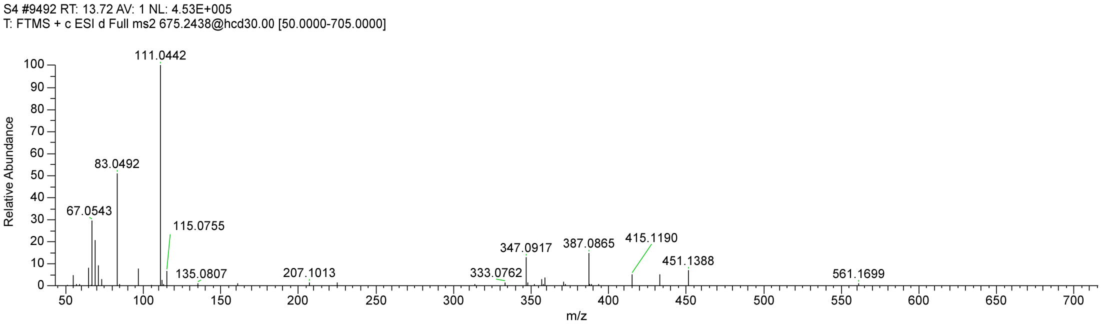

Figure S27. MS/MS spectrum of ion at *m/z* 675.2438 [(**11a**+H)$^+$].

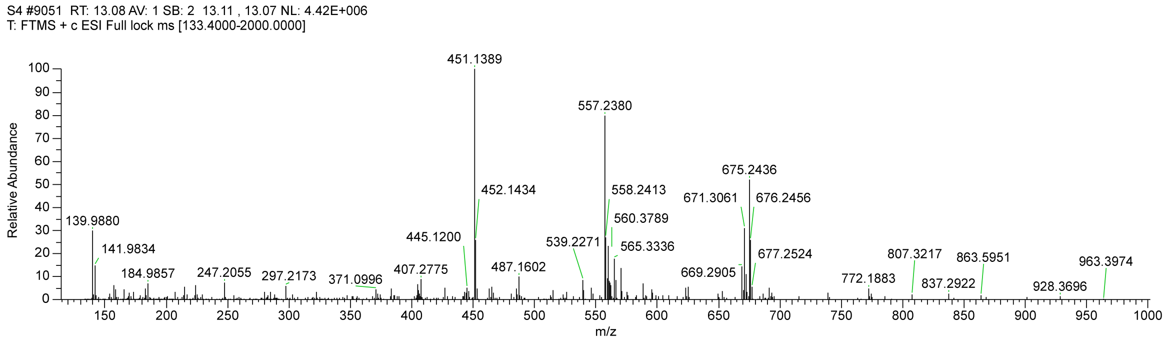

Figure S28. MS spectrum of compound **11b**.

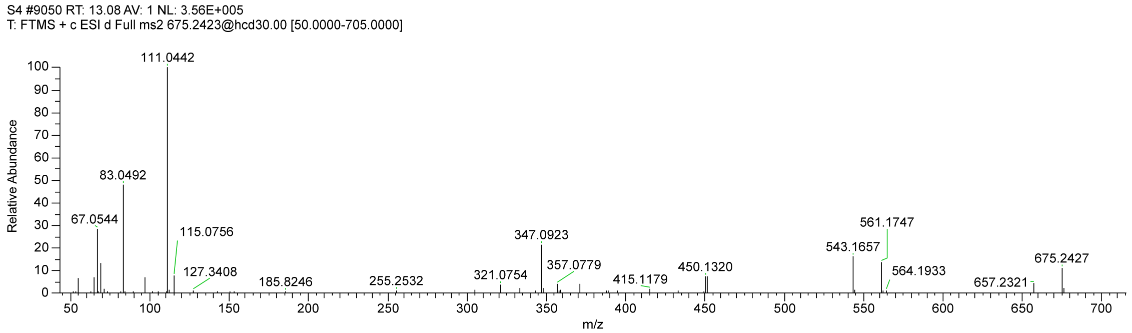

Figure S29. MS/MS spectrum of ion at *m/z* 675.2436 [(**11b**+H)$^{+}$].

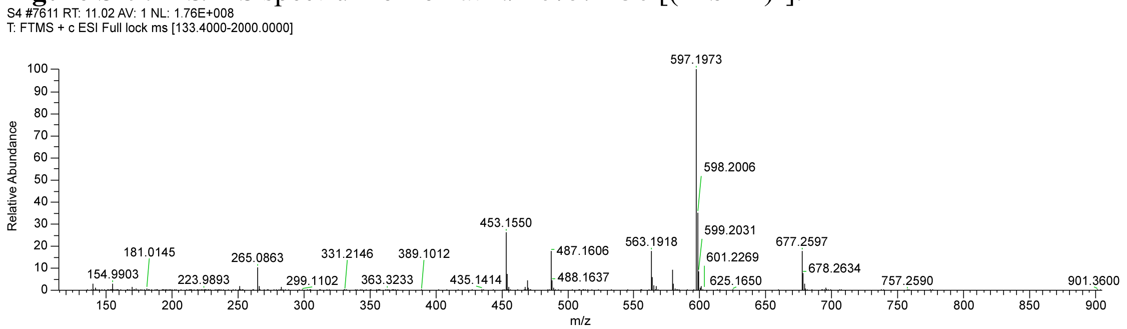

Figure S30. MS spectrum of compound **12**.

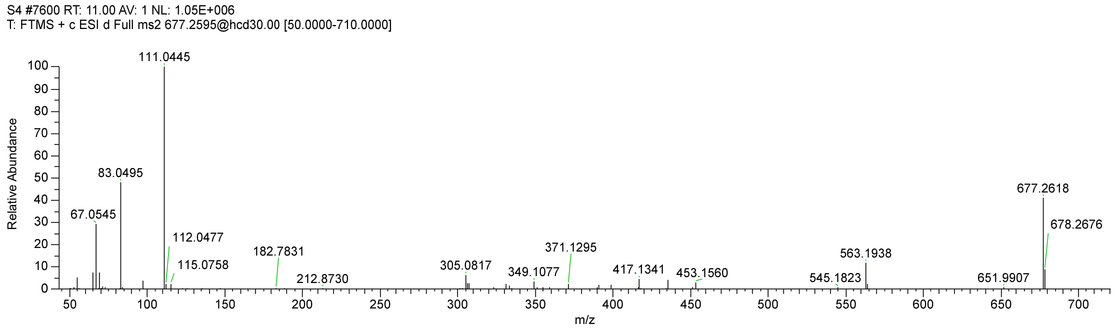

Figure S31. MS/MS spectrum of ion at *m/z* 677.2597 [(**12**+H)$^{+}$].

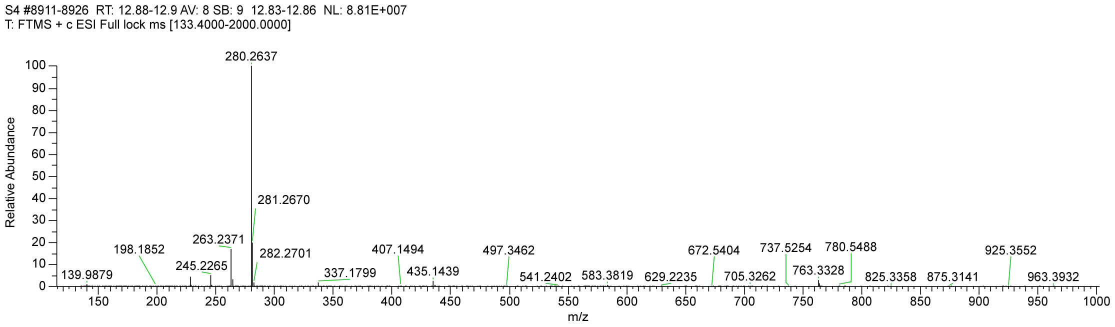

Figure S32. MS spectrum of compound **13**.

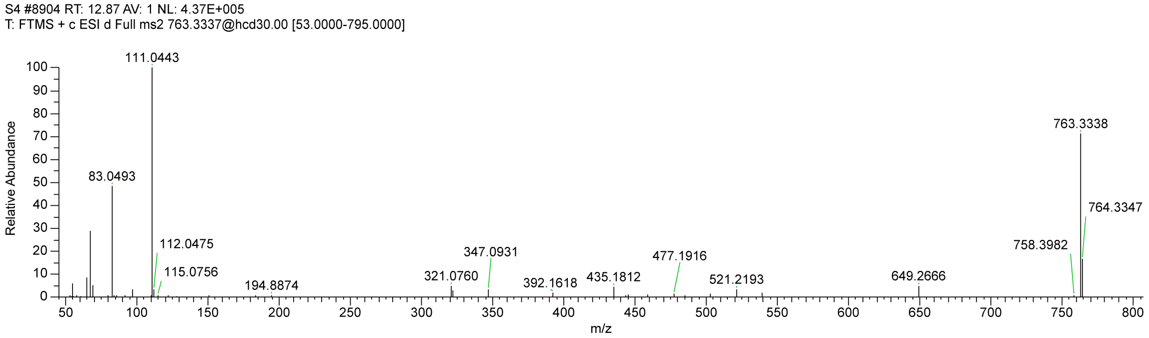

Figure S33. MS/MS spectrum of ion at *m/z* 763.3328 [(**13**+H)$^+$].

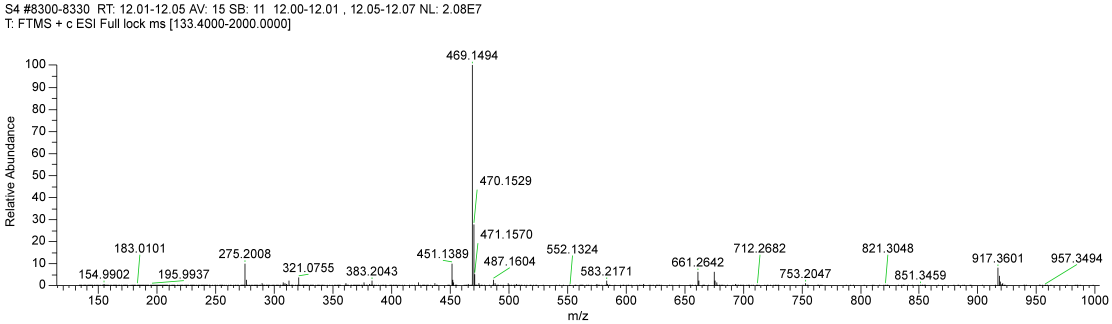

Figure S34. MS spectrum of compound **14**.

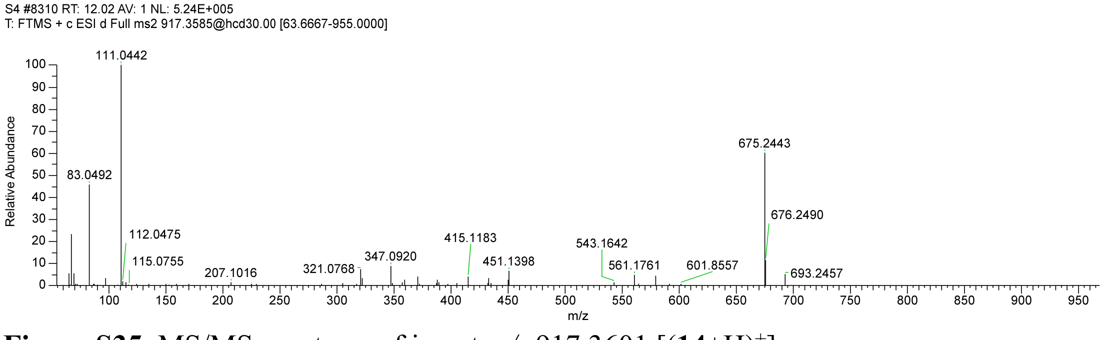

Figure S35. MS/MS spectrum of ion at *m/z* 917.3601 [(**14**+H)$^+$].

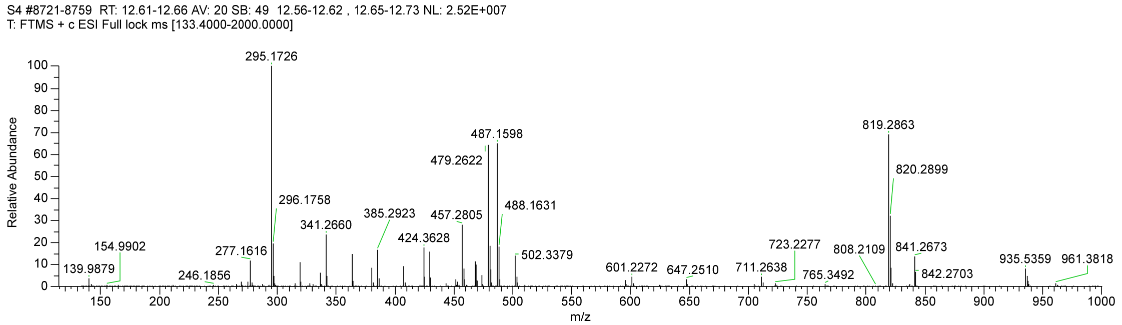

Figure S36. MS spectrum of compound **15a**.

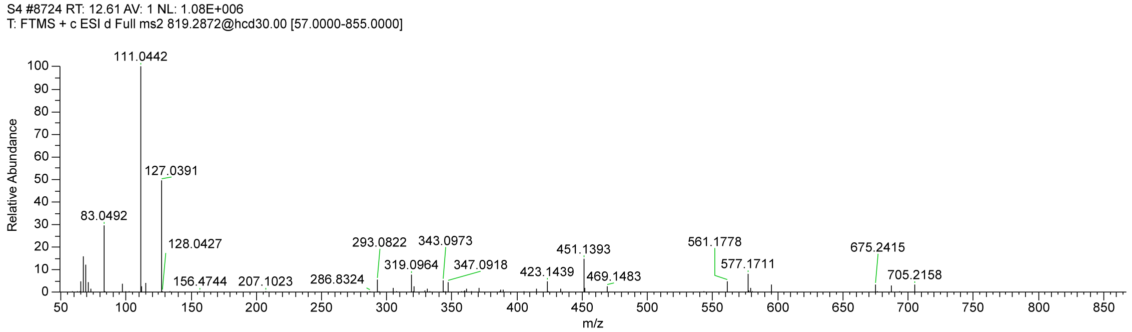

Figure S37. MS/MS spectrum of ion at m/z 819.2863 [(**15a**+H)+].

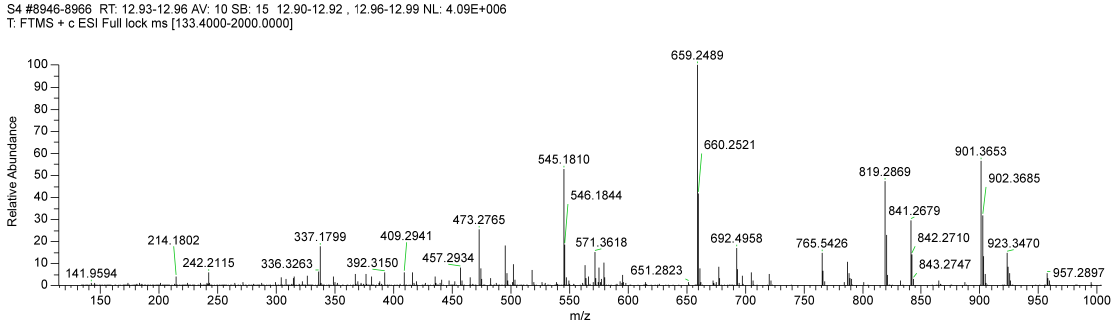

Figure S38. MS spectrum of compound **15b**.

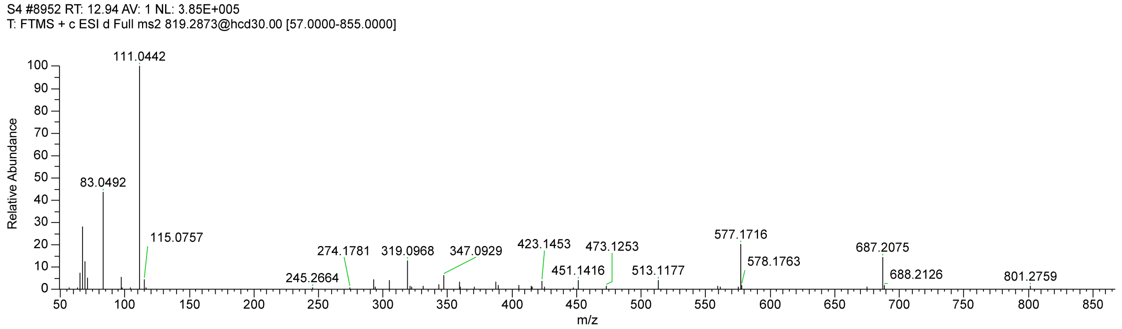

Figure S39. MS/MS spectrum of ion at *m/z* 819.2869 [(**15b**+H)$^+$].

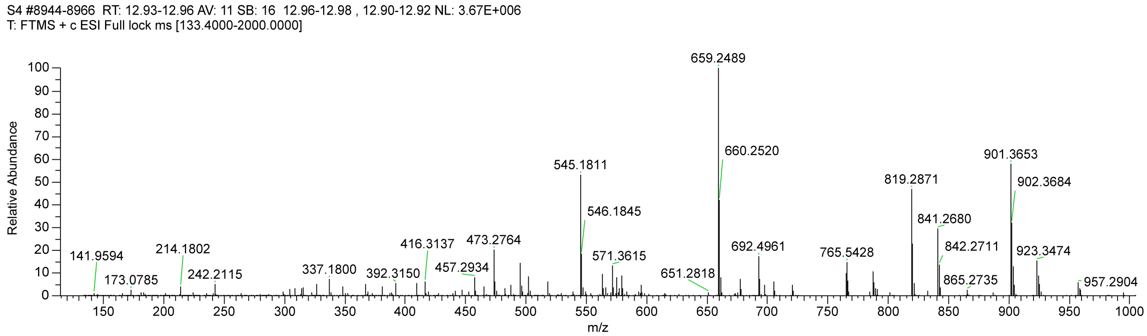

Figure S40. MS spectrum of compound **16**.

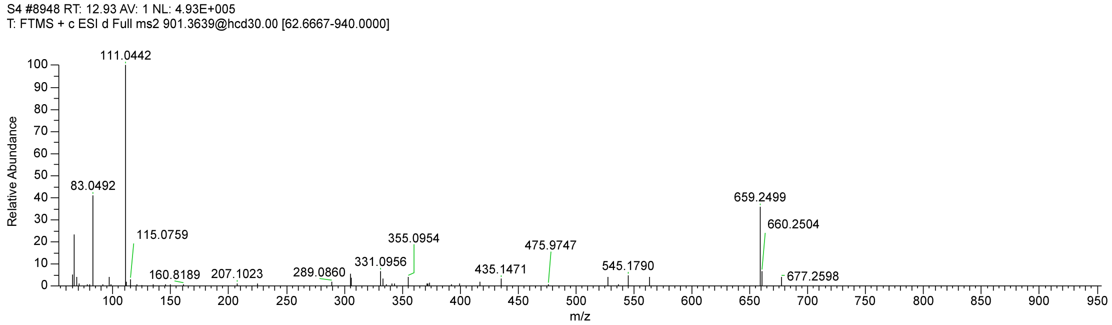

Figure S41. MS/MS spectrum of ion at *m/z* 901.3653 [(**16**+H)$^+$].

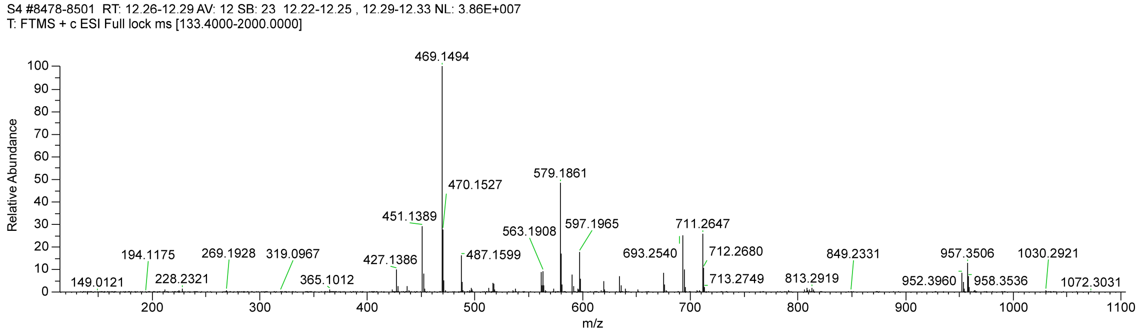

Figure S42. MS spectrum of compound **17**.

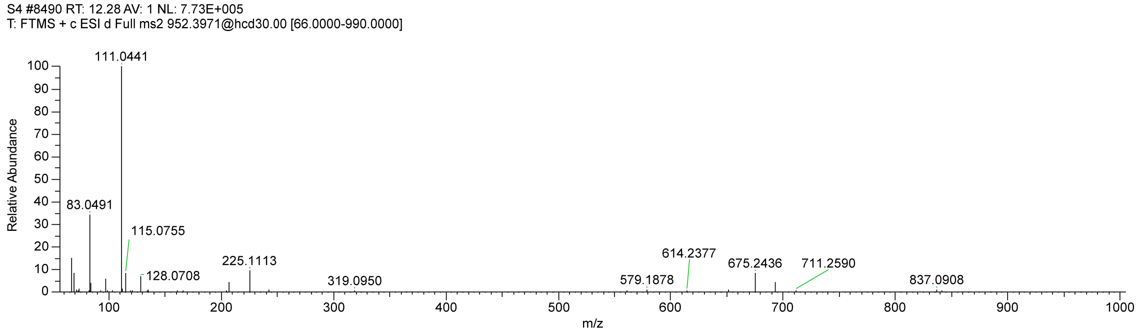

Figure S43. MS/MS spectrum of ion at *m/z* 952.3960 [(**17**+NH_4)$^+$].

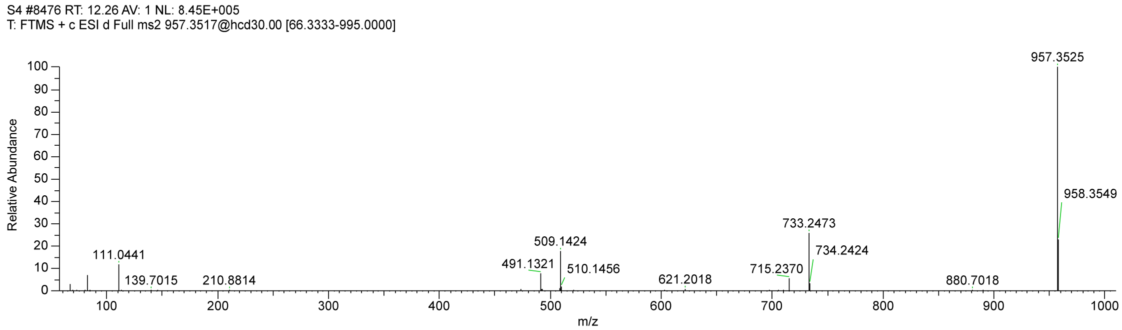

Figure S44. MS/MS spectrum of ion at *m/z* 957.3506 [(**17**+Na)$^+$].

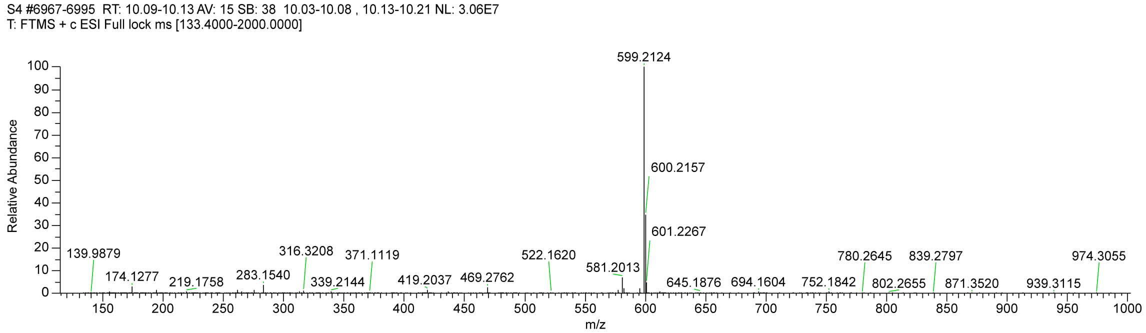

Figure S45. MS spectrum of compound **18a**.

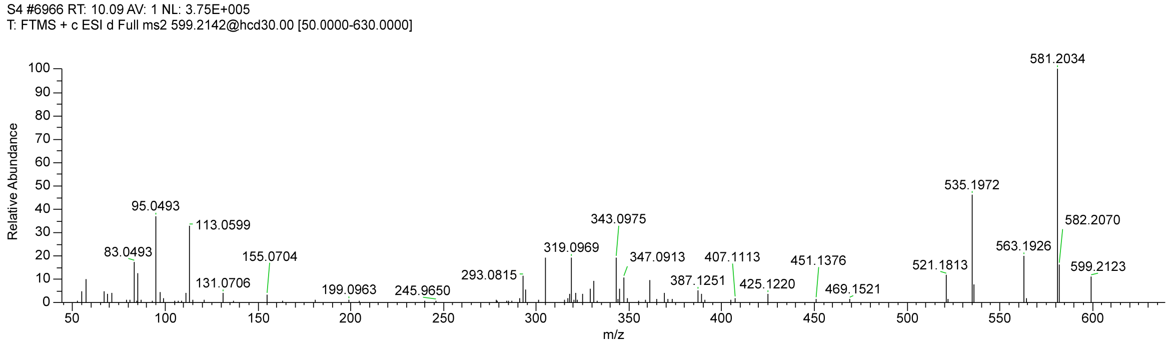

Figure S46. MS/MS spectrum of ion at *m/z* 599.2142 [(**18a**+H)$^+$].

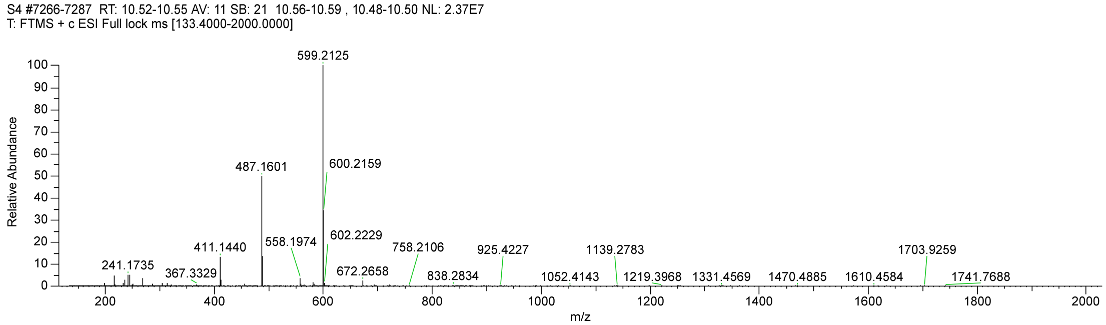

Figure S47. MS spectrum of compound **18b**.

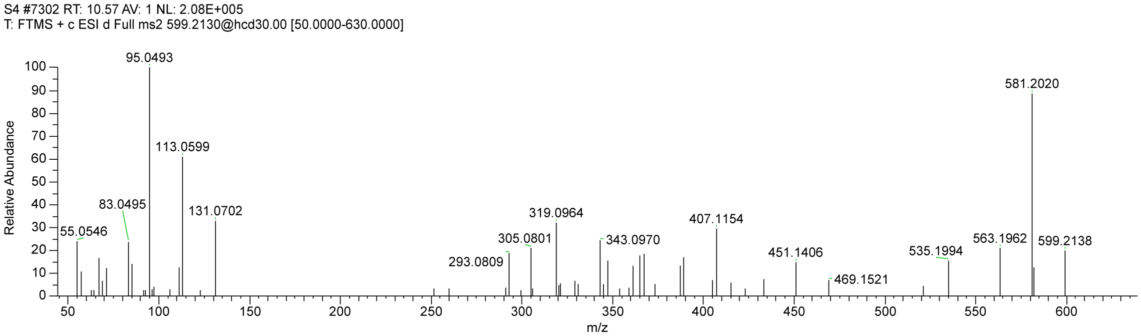

Figure S48. MS/MS spectrum of ion at *m/z* 599.2125 [(**18b**+H)$^+$].

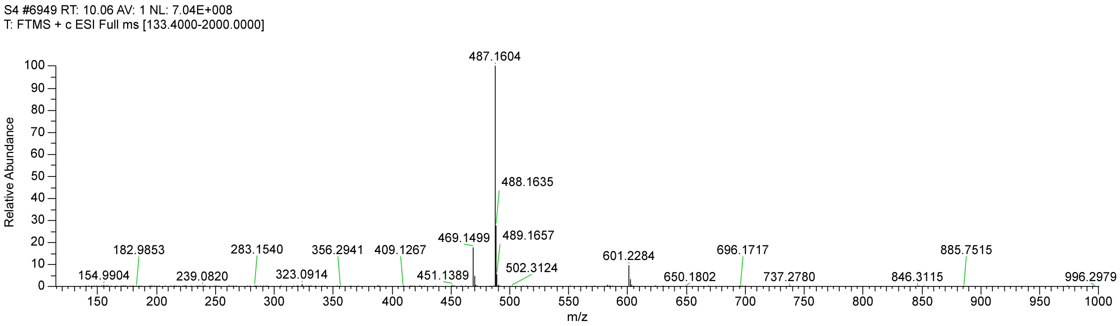

Figure S49. MS spectrum of compound **19a**.

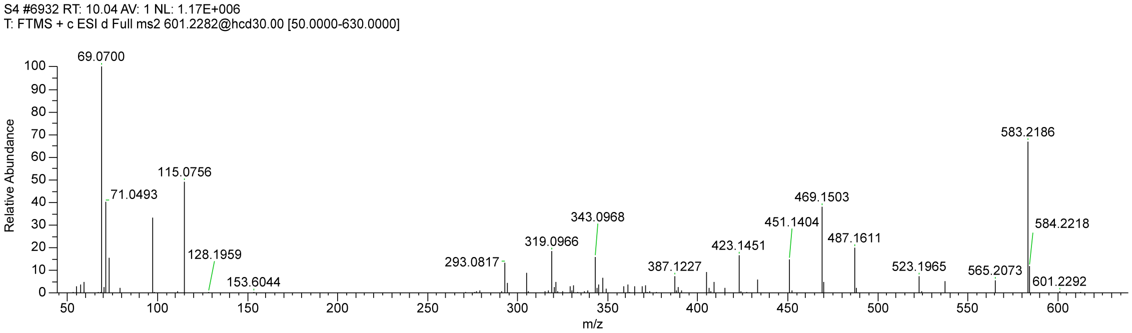

Figure S50. MS/MS spectrum of ion at *m/z* 601.2284 [(**19a**+H)$^+$].

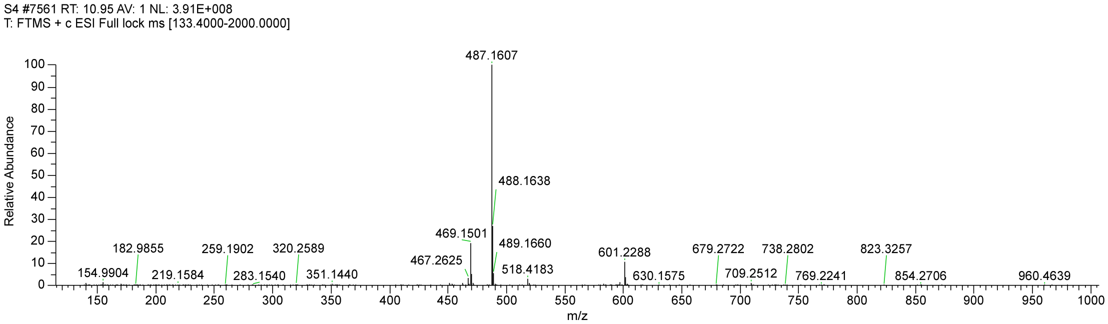

Figure S51. MS spectrum of compound **19b**.

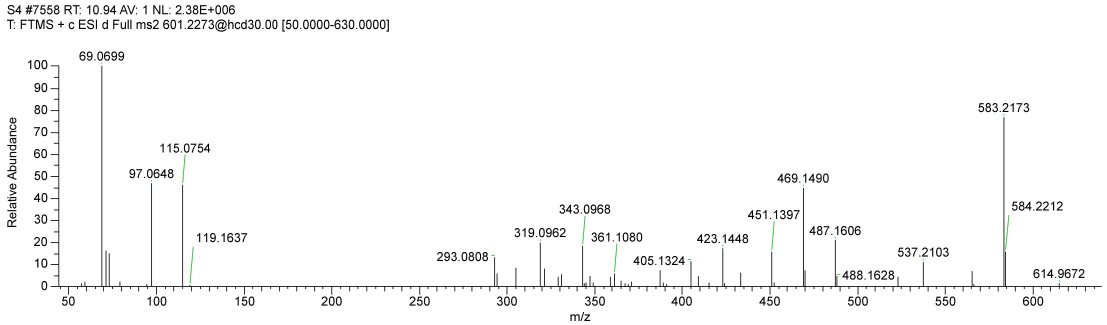

Figure S52. MS/MS spectrum of ion at *m/z* 601.2288 [(**19b**+H)$^{+}$].

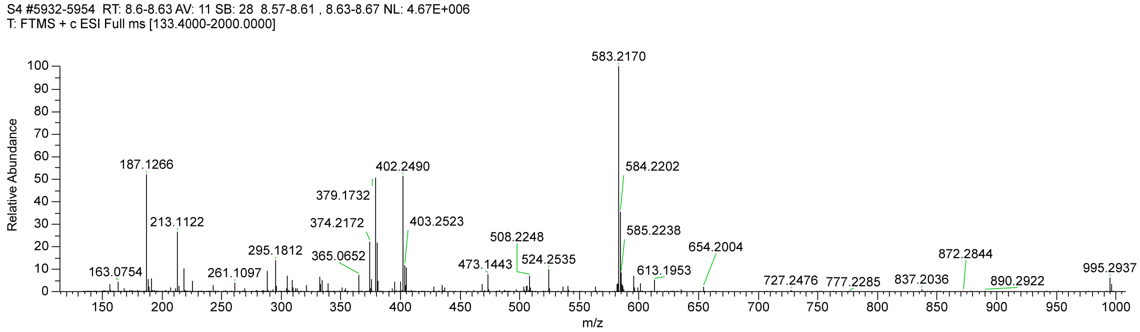

Figure S53. MS spectrum of compound **20**.

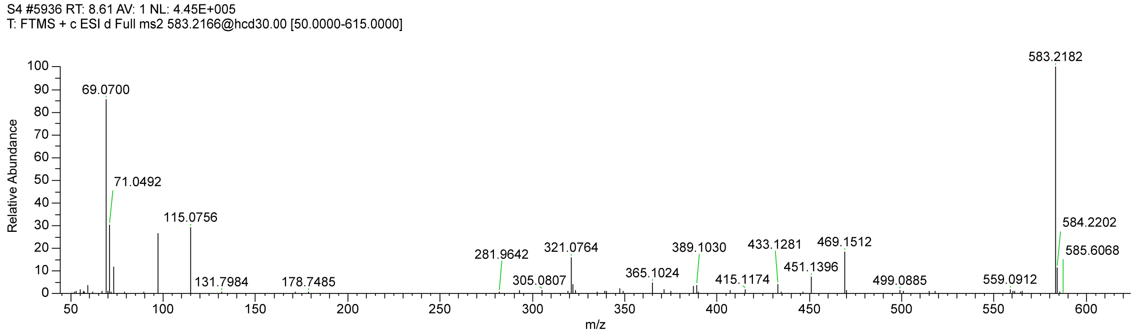

Figure S54. MS/MS spectrum of ion at *m/z* 583.2170 [(**20**+H)$^{+}$].

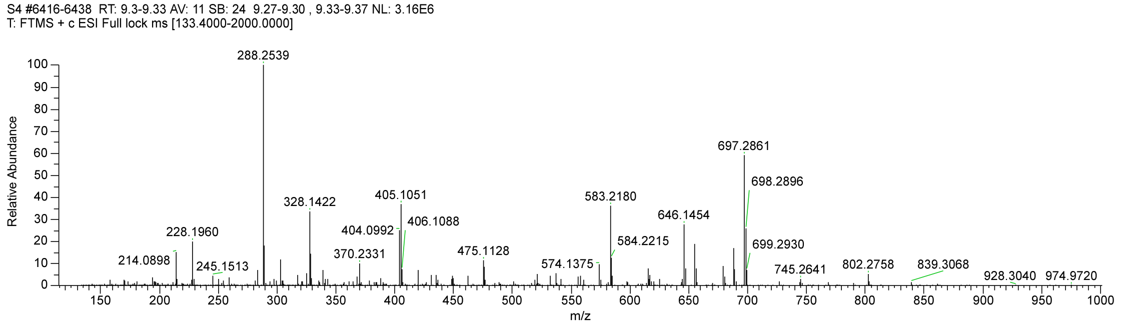

Figure S55. MS spectrum of compound **21**.

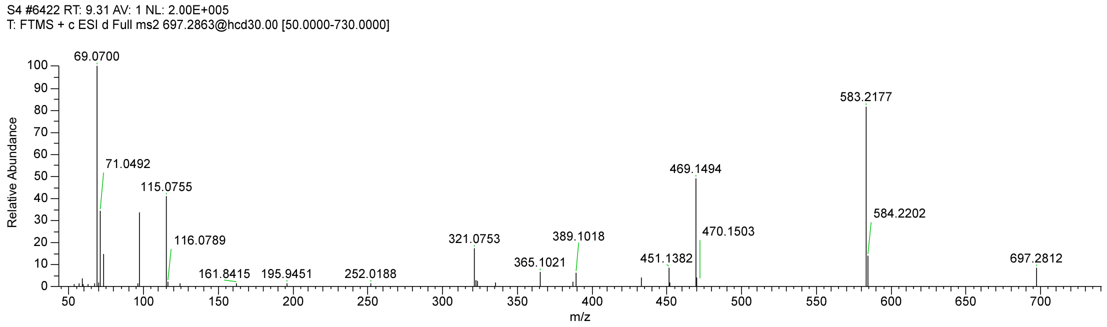

Figure S56. MS/MS spectrum of ion at *m/z* 697.2861 [(**21**+H)$^+$].

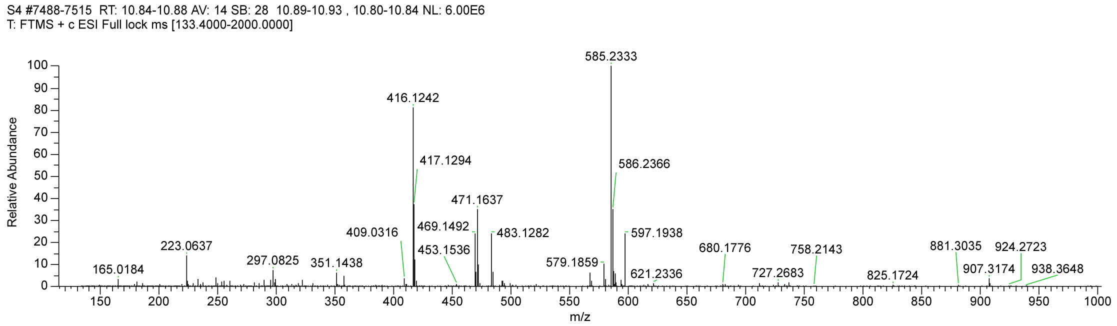

Figure S57. MS spectrum of compound **22**.

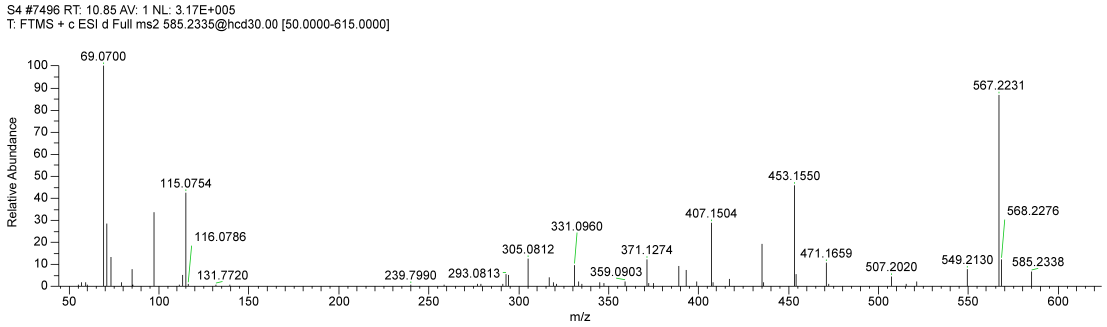

Figure S58. MS/MS spectrum of ion at *m/z* 585.2333 [(**22**+H)$^+$].

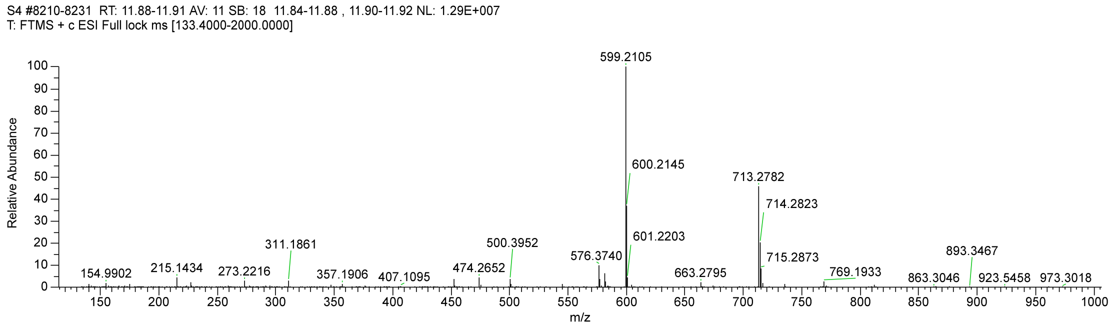

Figure S59. MS spectrum of compound **23**.

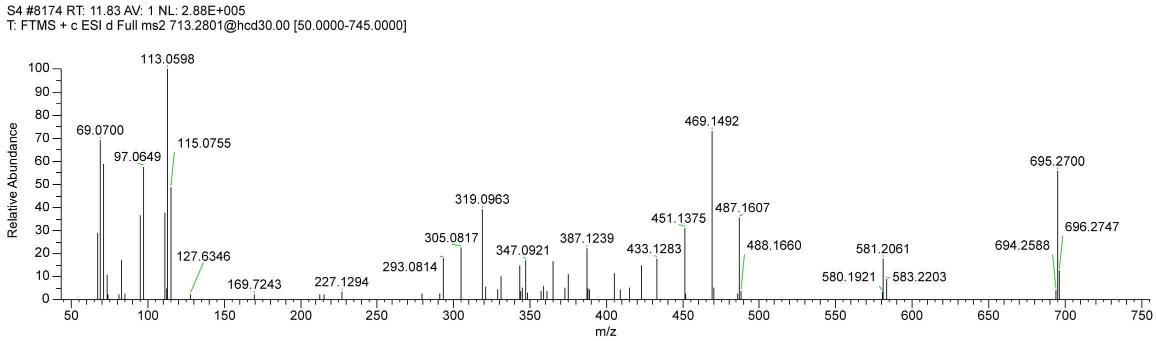

Figure S60. MS/MS spectrum of ion at *m/z* 713.2782 [(**23**+H)$^+$].

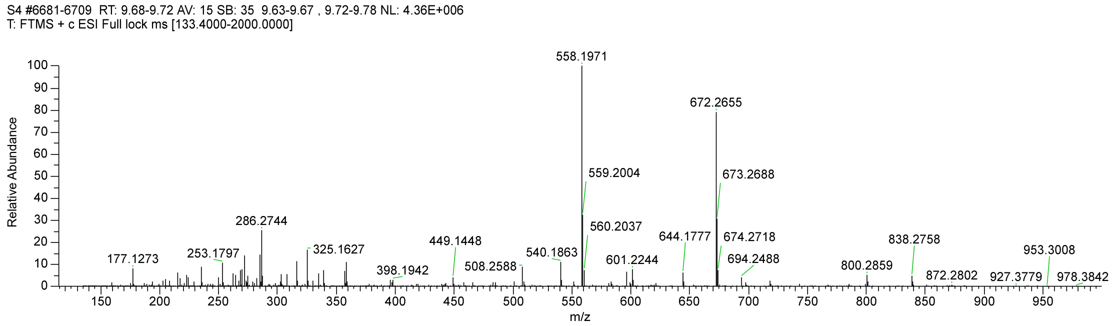

Figure S61. MS spectrum of compound **24**.

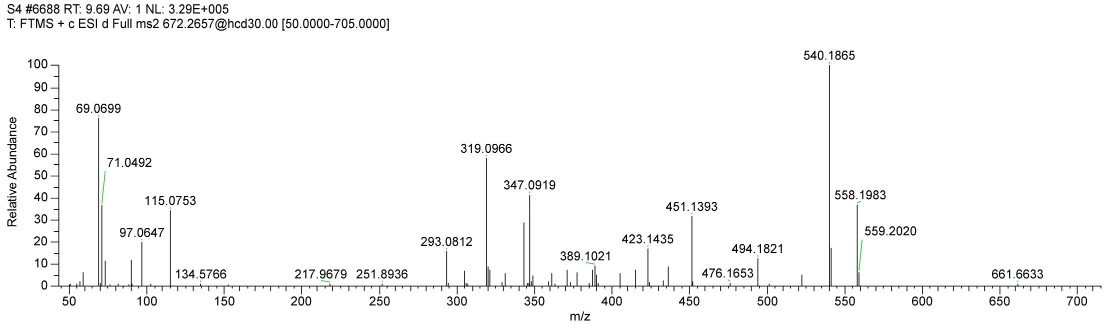

Figure S62. MS/MS spectrum of ion at *m/z* 672.2655 [(**24**+H)$^+$]

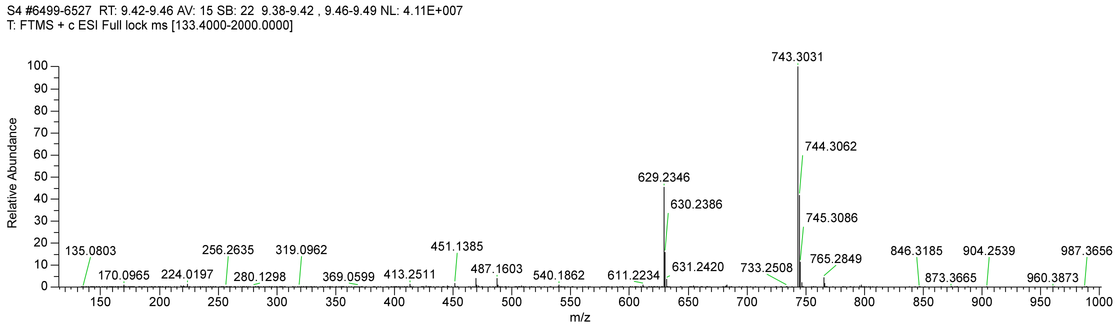

Figure S63. MS spectrum of compound **25**.

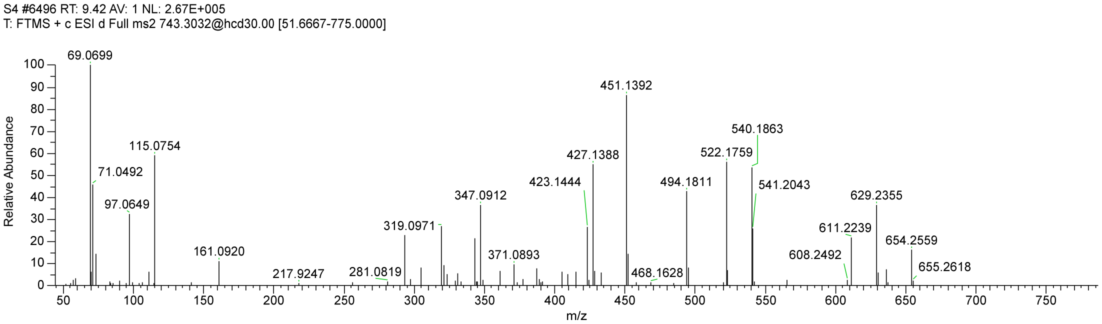

Figure S64. MS/MS spectrum of ion at *m/z* 743.3031 [(**25**+H)$^{+}$].

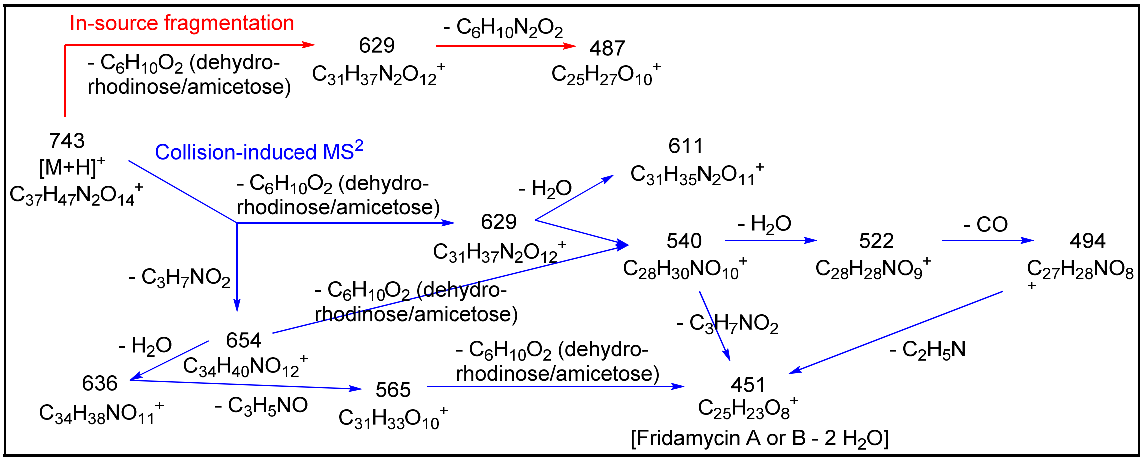

Figure S65. Fragmentation scheme for compound **25** from [M+H]$^{+}$ to the aglycone ion.

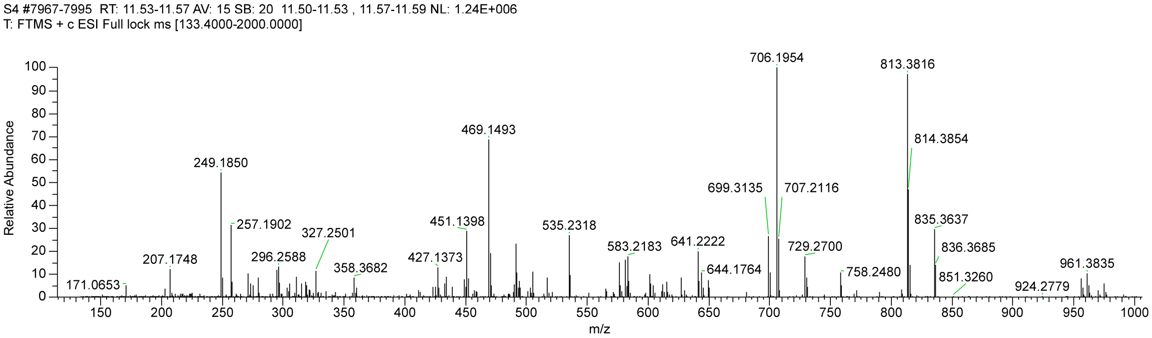

Figure S66. MS spectrum of compound **26**.

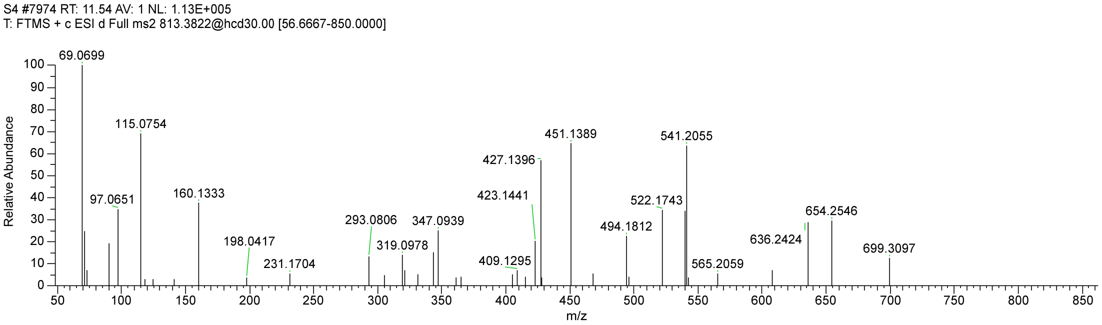

Figure S67. MS/MS spectrum of ion at *m/z* 813.3816 [(**26**+H)$^{+}$].

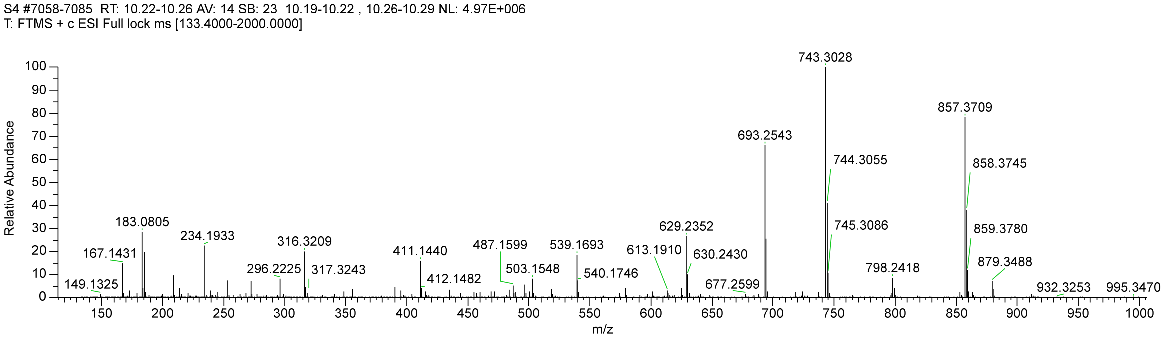

Figure S68. MS spectrum of compound **27**.

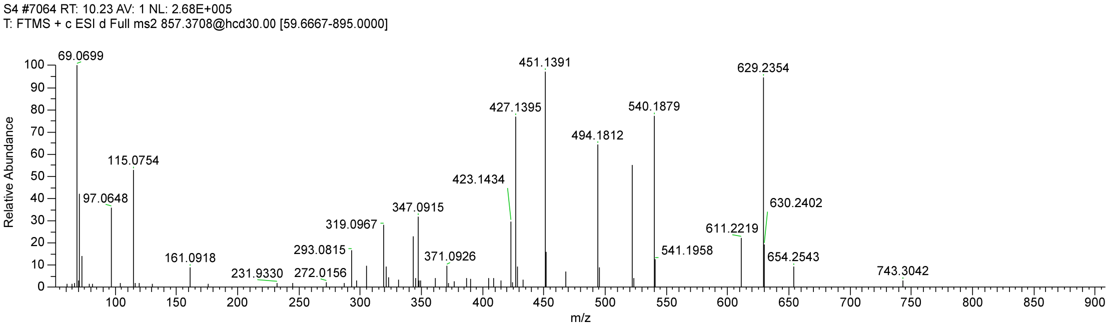

Figure S69. MS/MS spectrum of ion at *m/z* 857.3709 [(**27+H**)$^{+}$].

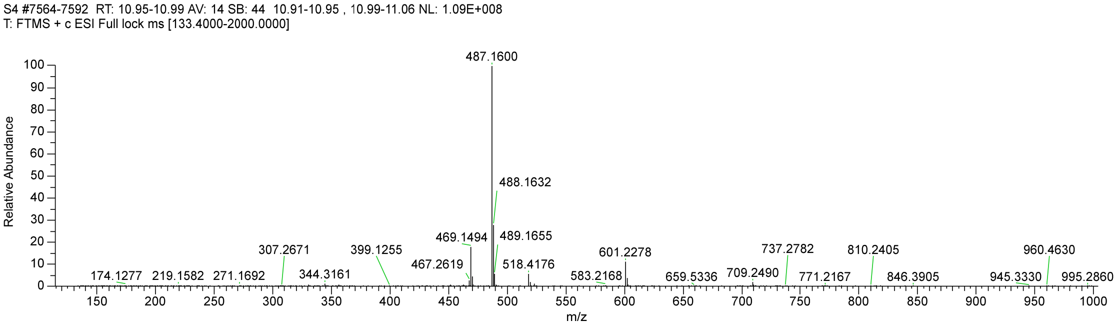

Figure S70. MS spectrum of compound **28**.

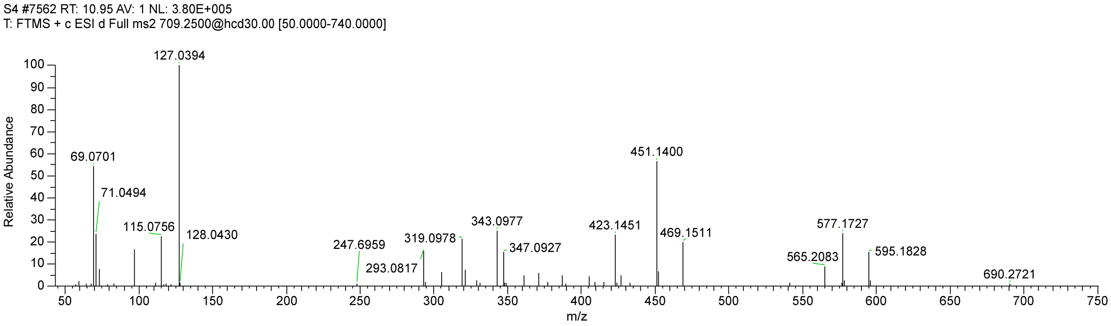

Figure S71. MS/MS spectrum of ion at *m/z* 709.2490 [(**28**+H)$^{+}$].

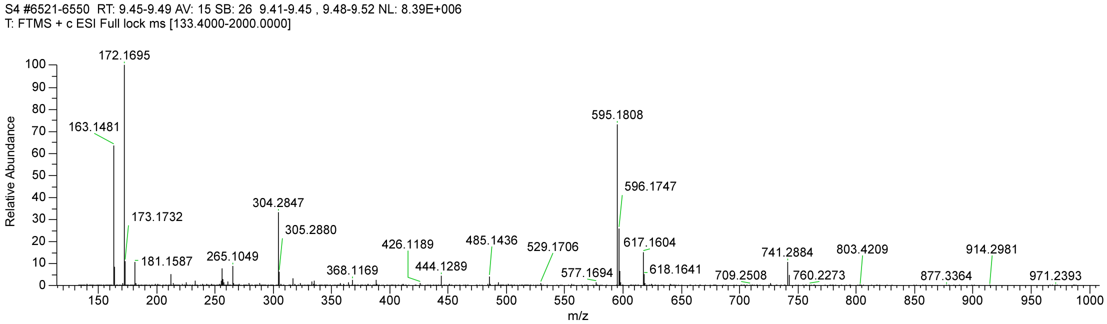

Figure S72. MS spectrum of compound **29**.

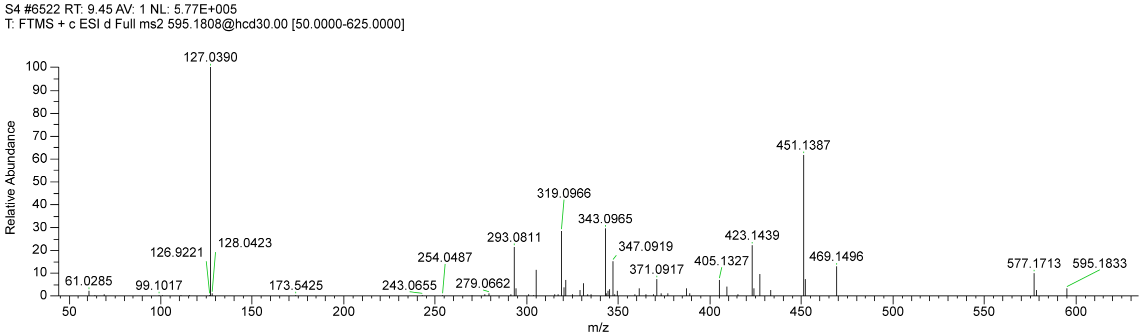

Figure S73. MS/MS spectrum of ion at *m/z* 595.1808 [(**29**+H)$^+$].

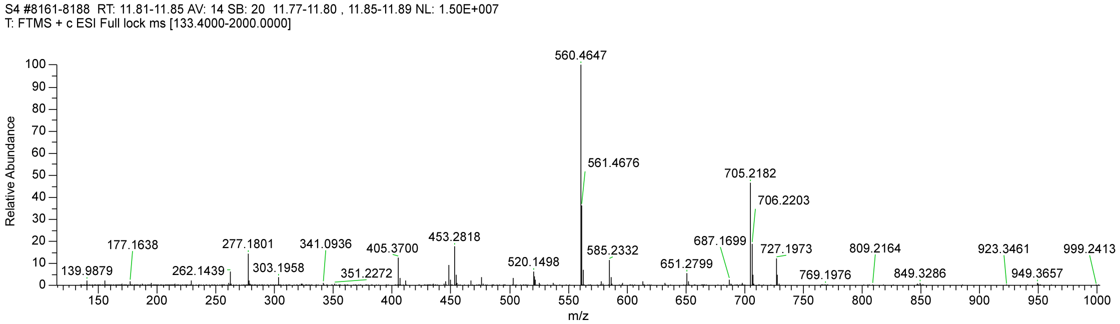

Figure S74. MS spectrum of compound **30**.

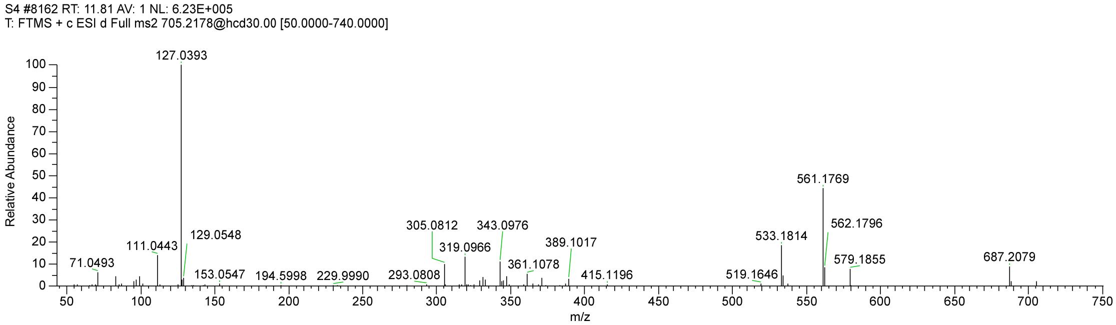

Figure S75. MS/MS spectrum of ion at *m/z* 705.2182 [(**30**+H)$^+$].

Table S2. Summary of antiSMASH 6.0 secondary metabolites predicted from the RO-S4 strain

Cluster no.	Metabolite class	Most similar known biosynthetic cluster (%)	Contig no.	Span [nt]	
				From	To
**Cluster 1**	Siderophore	Desferrioxamin B/ desferrioxamine E (83%)	ROS4_1	214,500	226,272
**Cluster 2**	T2PKS, Oligosaccharide Phenazine, Siderophore	grincamycin (97%)	ROS4_2	140,051	226,533
**Cluster 3**	Ectoine	ectoine (100%)	ROS4_4	76,930	87,328
**Custer 4**	Lanthipeptide-class-i	kanamycin (1%)	ROS4_5	124,831	150,105
**Cluster 5**	Terpene	geosmin (100%)	ROS4_ 6	126,687	148,843
**Cluster 6**	T2PKS	spore pigment (83%)	ROS4_10	11,361	83,870
**Cluster 7**	T3PKS	alkylresorcinol (100%)	ROS4_11	114,736	155,884
**Cluster 8**	Terpene	carotenoid (54%)	ROS4_16	35,638	59,717
**Cluster 9**	T1PKS	ashimides (12%)	ROS4_20 (1)	259	24,193
**Cluster 10**	RiPP-like, blactam, Terpene	Unknown	ROS4_20 (2)	24,913	58,437
**Cluster 11**	T1PKS	enduracidin (12%)	ROS4_20 (3)	71,642	115,844
**Cluster 12**	Phenazine	fredericamycin A (6%)	ROS4_21	49,206	69,667
**Cluster 13**	Terpene	albaflavenone (100%)	ROS4_26	49,303	70,316
**Cluster 14**	Terpene	hopene (92%)	ROS4_29	68,654	86,555
**Cluster 15**	NRPS, T1PKS, other	polyoxypeptin (48%)	ROS4_33	1	82,128
**Cluster 16**	Lanthipeptide-class-i	Unknown	ROS4_37	21,339	45,849
**Cluster 17**	NRPS, T1PKS	antimycin (100%)	ROS4_41	1	47,509
**Cluster 18**	RiPP-like	informatipeptin (42%)	ROS4_70	12,446	22,661
**Cluster 19**	Butyrolactone	griseoviridin / fijimycin A (11%)	ROS4_89	1	6,885

Table S3. Deduced functions of the ORFs in the ROS4_2 cluster '*Streptomyces* sp. RO-S4 strain and their BLASTs. We have considered only the sequences that showed a homology >50%

ORF	Gene annotation	Putative function	Length (nt)	Most similar protein/ Identity[a]	Strain	Accession Number
**1**	LRR80_00441	Carboxymuconolactone decarboxylase	1302	forQ, 80.93%<br>Homolog in rubrolone, 82.04%	*Streptomyces sp. KY5*<br>*Streptomyces sp. KIB-H033*	AQP25549.1<br>AOZ61217.1
**2**	LRR80_00442	Hypothetical protein	993	n.a.		
**3**	LRR80_00443	Endoribonuclease	396	n.a.		
**4**	LRR80_ 00444	ATPase	1380	n.a.		
**5**	LRR80_ 00445	Phenazine biosynthesis-like protein	858	cnq727, 62.54%	*Streptomyces sp. CNQ-509*	AIT42117.1
**6**	LRR80_ 00446	Asparaginase	1677	n.a.		
**7**	LRR80_00447	Hypothetical protein unknown function	486	n.a.		
**8**	LRR80_00448	Putative methyltransferase	1011	Homolog in tetarimycin A, 60.90%	*uncultured bacterium*	AFY23025.1
**9**	LRR80_00449	3-demethylubiquinone-9 3-methyltransferase	447	Homolog in SW-163C, 55.56%	*Streptomyces sp. SNA15896*	BAI63271.1
**10**	LRR80_00450	Phenazine biosynthesis protein A/B	492	Homolog in tetarimycin A, 74.66%<br>Homolog in streptophenazine B, 69.18%	*uncultured bacterium*<br>*Streptomyces sp. CNB091*	AFY23026.1<br>WP_018960032.1
**11**	LRR80_00451	Asparagine synthase	1857	PieD, 61.39%<br>ttmN, 60.84%	*Streptomyces sp. SCSIO 03032*<br>*Streptomyces afghaniensis*	AHE80999.1<br>ALJ49932.1
**12**	LRR80_00452	Pyridoxamine 5'-phosphate oxidase	621	LphzG, 53.93%	*Streptomyces lomondensis*	AKC91617.1
**13**	LRR80_00453	Hypothetical protein	348	Homolog in tetarimycin A ,61.05%	*uncultured bacterium*	AFY23027.1

**14**	LRR80_00454	Isochorismate synthase	1905	stnM1, 62.62%<br>Homolog in diazaquinomycin A, 61.28%	*Streptomyces flocculus*<br>*Streptomyces sp. F001*	AFW04564.1<br>RZB16709.1
**15**	LRR80_00455	Isochorismatase	624	stnM2, 67.80%<br>Homolog, 68.14%	*Streptomyces flocculus*<br>*uncultured bacterium*	AFW04565.1<br>AFY23028.1
**16**	LRR80_00456	3-deoxy-7-phosphoheptulonate synthase activity	1125	stnN, 64.72%<br>StrepF001_25935, 63.29%<br>epaO, 63.97%	*Streptomyces flocculus*<br>*Streptomyces sp. F001*<br>*Kitasatospora sp. HKI 714*	AFW04567.1<br>RZB16706.1<br>AHW81474.1
**17**	LRR80_00457	Hypothetical protein	648	n.a.		
**18**	LRR80_00458	Hypothetical protein	375	n.a.		
**19**	LRR80_00459	Regulatory protein	366	Homolog in bleomycin, 84.69%	*Streptomyces verticillus*	AAG02351.1
**20**	LRR80_00460	Hypothetical protein	2415	n.a.		
**21**	LRR80_00461	SNF2 Helicase protein	2868	Homolog in kosinostatin, 58.17%	*Micromonospora sp. TP-A0468*	AFJ52712.1
**22**	LRR80_00462	Hypothetical protein	606	n.a.		
**23**	LRR80_00463	Putative glucokinase	1149	n.a.		
**24**	LRR80_00464	Regulatory protein	738	n.a.		
**25**	LRR80_00465	Oxidoreductase	1023	n.a.		
**26**	LRR80_00466	Thioesterase superfamily protein	435	n.a.		
**27**	LRR80_00467	LuxR family DNA-binding response regulator	675	lipReg1, 85.00%	*Kitasatospora aureofaciens*	ABB05090.1
**28**	LRR80_00468	Histidine kinase	1218	lipReg25, 75.93%	*Kitasatospora aureofaciens*	ABB05091.1
**29**	LRR80_00469	Hypothetical protein	204	n.a.		
**30**	LRR80_00470	Ribonuclease	429	Pau53, 79.21%<br>lipX1, 66.90%	*Streptomyces Paulus*<br>*Kitasatospora aureofaciens*	AIE54220.1<br>ABB05092.1
**31**	LRR80_00471	Dehydrogenase/reductase SDR	753	Homolog in ebelactone, 59.52%	*Kitasatospora aburaviensis*	SCN11974.1

**32**	LRR80_00472	Allantoicase	1116	n.a.		
**33**	LRR80_00473	Amidohydrolase	1341	PauY52, 77.53%	*Streptomyces sp. YN86*	AIE54272.1
**34**	LRR80_00474	IclR family transcriptional regulator	807	PauY51, 86.96%	*Streptomyces sp. YN86*	AIE54271.1
**35**	LRR80_00475	Hypothetical protein	312	PauY50, 56.38%	*Streptomyces sp. YN86*	AIE54270.1
**36**	LRR80_00476	NTP transferase	600	Orf-3, 86.43%	*Streptomyces lusitanus*	AGO50601.1
**37**	LRR80_00477	Malate synthase	1626	Orf-2, 94.27%<br>PauY45, 84.62%	*Streptomyces lusitanus*<br>*Streptomyces sp. YN86*	AGO50602.1<br>AIE54265.1
**38**	LRR80_00478	SelT/SelW/SelH family protein	279	n.a.		
**39**	LRR80_00479	Hypothetical protein	924	Orf-1, 93.81%	*Streptomyces lusitanus*	AGO50603.1
**40**	LRR80_00480	NADH: flavin oxidoreductase / NADH oxidase	1119	GcnA, 95.70%<br>spr3, 85.75 %	*Streptomyces lusitanus*<br>*Streptomyces sp. TK08046*	AGO50604.1<br>BAV16992.1
**41**	LRR80_00481	Drug resistance transporter	1569	GcnB, 92.87%<br>sqnB, 83.23%	*Streptomyces lusitanus*<br>*Streptomyces sp.*	AGO50605.1<br>ARO44650.1
**42**	LRR80_00482	NADPH-dependent FMN reductase	600	GcnC, 94.47%<br>sqnC, 85.43%<br>spr5, 85.93%	*Streptomyces lusitanus*<br>*Streptomyces sp.*<br>*Streptomyces sp. TK08046*	AGO50606.1<br>ARO44679.1<br>BAV16994.1
**43**	LRR80_00483	Regulatory proteins	738	GcnD, 94.69%<br>sqnD, 83.67 %	*Streptomyces lusitanus*<br>*Streptomyces sp.*	AGO50607.1<br>ARO44676.1
**44**	LRR80_00484	Hypothetical protein	843	GcnE, 80.71%<br>sprA, 71.73%	*Streptomyces lusitanus*<br>*Streptomyces sp. TK08046*	AGO50608.1<br>BAV16996.1

**45**	LRR80_00485	Monooxygenase FAD-binding	1488	GcnF, 95.55%	*Streptomyces lusitanus*	AGO50609.1
				UrdE, 91.50%	*Streptomyces fradiae*	CAA60567.1
				sqnF, 89.68%	*Streptomyces sp.*	ARO44651.1
				schP10 86.76%	*Streptomyces sp. SCC 2136*	CAH10119.1
				PgaE, 75.82%	*Streptomyces sp. PGA64*	AAK57522.1
				lanE, 73.17%	*Streptomyces cyanogenus*	AAD13534.1
				bexE, 73.12%	*Amycolatopsis orientalis subsp. vinearia*	ADI71441.1
				ovmOI, 69.92%	*Streptomyces antibioticus*	ARK36153.1
				kinO2, 61.72%	*Streptomyces murayamaensis*	AAO65352.1
**46**	LRR80_00486	Polyketide synthesis cyclase	327	sprC, 96.26%	*Streptomyces sp. TK08046*	BAV16998.1
				sqnBB, 90.74%	*Streptomyces sp.*	ARO44683.1
				schP9, 90.65%	*Streptomyces sp. SCC 2136*	CAH10118.1
**47**	LRR80_00487	Beta-ketoacyl synthase	1281	GcnH, 98.12%	*Streptomyces lusitanus*	AGO50610.1
				sprD, 93.66%	*Streptomyces sp. TK08046*	BAV16999.1
				sqnH, 91.61%	*Streptomyces sp.*	ARO44655.1
				SchP8, 89.86%	*Streptomyces sp. SCC 2136*	CAH10117.1
				UrdA, 92.49%	*Streptomyces fradiae*	CAA60569.1
**48**	LRR80_00488	CLF Beta-ketoacyl synthase	1221	GcnI, 95.56%	*Streptomyces lusitanus*	AGO50611.1
				sprE, 90.42%	*Streptomyces sp. TK08046*	BAV17000.1
				sqnI, 86.70%	*Streptomyces sp.*	ARO44656.1
				OvmK, 74.57%	*Streptomyces antibioticus*	ARK36156.1
				URdB, 86.70%	*Streptomyces fradiae*	CAA60570
**49**	LRR80_00489	Acyl carrier protein	270	GcnJ, 98.88%	*Streptomyces lusitanus*	AGO50612.1
				sqnJ, 85.39%	*Streptomyces sp.*	ARO44684.1
				sprF, 84.27%	*Streptomyces sp. TK08046*	BAV17001.1
**50**	LRR80_00490	Dehydrogenase/reductase SDR	786	GcnK, 93.49 %	*Streptomyces lusitanus*	AGO50613.1
				sprG, 94.25%	*Streptomyces sp. TK08046*	BAV17002.1
				sqnK, 91.95%	*Streptomyces sp.*	ARO44671.1
				schP5, 88.63%	*Streptomyces sp. SCC 2136*	CAH10114.1

**51**	LRR80_00491	Cyclase/Dehydrase	936	GcnL, 93.89%	*Streptomyces lusitanus*	AGO50614.1
				sprH, 91.00%	*Streptomyces sp. TK08046*	BAV17003.1
				UrdL, 86.17%	*Streptomyces fradiae*	AAF00205.1
				PgaL, 79.48%	*Streptomyces sp. PGA64*	AAK57529.1
				May12, 77.17%	*Streptomyces sp.*	AVO00811.1
**52**	LRR80_00492	Monooxygenase FAD-binding	1989	GcnM, 90.63%	*Streptomyces lusitanus*	AGO50615.1
				SprI, 87.01%	*Streptomyces sp. TK08046*	BAV17004.1
				UrdM, 79.82%	*Streptomyces fradiae*	AFU51427.1
				sqnM, 82.86%	*Streptomyces sp.*	ARO44645.1
				schP3, 69.55%	*Streptomyces sp. SCC 2136*	CAH10112.1
				Homolog in Baikalomycyin, 74.66%	*Streptomyces sp. IB201691-2A2*	TRO56982.1
				pgaM, 75.00%	*Streptomyces sp. PGA64*	AAK57530.1
				Homolog in Fridamycin A, 57.43%	*Actinomadura sp. RB99*	MBD2892419.1
				ovmOII, 60.21%	*Streptomyces antibioticus*	CAG14970.1
				bexM, 65.65%	*Amycolatopsis orientalis subsp. Vinearia*	ADI71448.1
				SPW_4233, 64.90%	*Streptomyces sp. W007*	EHM27510.1
**53**	LRR80_00493	Drug resistance transporter, EmrB/QacA	1239	GcnN, 90.26%	*Streptomyces lusitanus*	AGO50616.1
				SprJ, 83.21%	*Streptomyces sp. TK08046*	BAV17005.1
				saqN, 81.04%	*Streptomyces sp.*	ARO44657.1
**54**	LRR80_00494	Glycosyltransferase	1293	GcnG1, 96.98%	*Streptomyces lusitanus*	AGO50617.1
				sqnG1, 88.14%	*Streptomyces sp.*	ARO44654.1
				sprGT1, 86.74%	*Streptomyces sp. TK08046*	BAV17006.1
				SchS10, 81.63%	*Streptomyces sp. SCC 2136*	CAH10110.1
**55**	LRR80_00495	Glycosyltransferase	1197	GcnG2, 92.46 %	*Streptomyces lusitanus*	AGO50618.1
				sprGT2, 83.92%	*Streptomyces sp. TK08046*	BAV17007.1
				sqnG2, 81.91 %	*Streptomyces sp.*	ARO44658.1
				SchS9, 74.74%	*Streptomyces sp. SCC 2136*	CAH10109.1
**56**	LRR80_00496	dTDP-4-dehydrorhamnose 3,5-epimerase	582	GcnS1, 97.56%	*Streptomyces lusitanus*	AGO50619.1
				sprK, 89.84%	*Streptomyces sp. TK08046*	BAV17008.1
				sqnS1, 92.02%	*Streptomyces sp.*	ARO44680.1

**57**	LRR80_00497	Glycosyltransferase	1131	GcnG3, 93.62%<br>sprGT3, 89.89%<br>sqnG3, 86.44%<br>schS7, 78.13%<br>UrdGT2, 77.81%	*Streptomyces lusitanus*<br>*Streptomyces sp. TK08046*<br>*Streptomyces sp.*<br>*Streptomyces sp. SCC 2136*<br>*Streptomyces fradiae*	AGO50620.1<br>BAV17009.1<br>ARO44660.1<br>CAF31363.2<br>AAF00209.1
**58**	LRR80_00498	Nucleotidyl transferase	1068	GcnS2, 95.21%<br>sprL, 92.11%<br>sqnS2, 89.86%	*Streptomyces lusitanus*<br>*Streptomyces sp. TK08046*<br>*Streptomyces sp.*	AGO50621.1<br>BAV17010.1<br>ARO44662.1
**59**	LRR80_00499	NAD dependent epimerase/dehydratase	984	GcnS3, 98.17%<br>sprM, 93.56%<br>sqnS3, 92.97%<br>schS5, 88.65%	*Streptomyces lusitanus*<br>*Streptomyces sp. TK08046*<br>*Streptomyces sp.*<br>*Streptomyces sp. SCC 2136*	AGO50622.1<br>BAV17011.1<br>ARO44664.1<br>CAF31365.1
**60**	LRR80_00500	NAD dependent epimerase/dehydratase	1059	GcnS4, 88.43%<br>sprN, 73.02%<br>sqnS4, 79.08%<br>schS4, 61.76%	*Streptomyces lusitanus*<br>*Streptomyces sp. TK08046*<br>*Streptomyces sp.*<br>*Streptomyces sp. SCC 2136*	AGO50623<br>BAV17012.1<br>ARO44667.1<br>CAF31366.1
**61**	LRR80_00501	DegT/DnrJ/EryC1/StrS aminotransferase	1311	GcnS5, 97.24%<br>sprO, 93.12%<br>sqnS5, 92.40%<br>urdQ, 88.71%<br>schS3, 88.94%<br>lanQ, 83.41%<br>aur1Q, 82.41%<br>saqQ, 80.46%<br>lct46, 81.92%	*Streptomyces lusitanus*<br>*Streptomyces sp. TK08046*<br>*Streptomyces sp.*<br>*Streptomyces fradiae*<br>*Streptomyces sp. SCC 2136*<br>*Streptomyces cyanogenus*<br>*Streptomyces aureofaciens*<br>*Micromonospora sp. Tu 6368*<br>*Streptomyces rishiriensis*	AGO50624.1<br>BAV17013.1<br>ARO44653.1<br>AAF72550.1<br>CAF31367.1<br>AAD13547.1<br>ACK77744.1<br>ACP19374.1<br>ABX71129.1

**62**	LRR80_00502	NAD dependent epimerase/dehydratase	765	GcnS6, 92.91%<br>sprP, 88.98%<br>urdR, 78.86%<br>sqnS6, 84.25%<br>lanR, 73.06%<br>PgaR, 70.66%	*Streptomyces lusitanus*<br>*Streptomyces sp. TK08046*<br>*Streptomyces fradiae*<br>*Streptomyces sp.*<br>*Streptomyces cyanogenus*<br>*Streptomyces sp. PGA64*	AGO50625.1<br>BAV17014.1<br>AAF72551.1<br>ARO44673.1<br>AAD13548.1<br>AHW57781.1
**63**	LRR80_00503	Hypothetical protein	954	GcnO, 86.75%<br>sqnO, 74.92%<br>sprQ, 77.70%	*Streptomyces lusitanus*<br>*Streptomyces sp.*<br>*Streptomyces sp. TK08046*	AGO50626.1<br>ARO44665.1<br>BAV17015.1
**64**	LRR80_00504	NDP-hexose 2,3-dehydratase	1404	GcnS7, 94.00%<br>sprR, 87.37%<br>sqnS7, 85.81%<br>schS2, 84.29%<br>urdS, 78.52%<br>lanS, 74.50%	*Streptomyces lusitanus*<br>*Streptomyces sp. TK08046*<br>*Streptomyces sp.*<br>*Streptomyces sp. SCC 2136*<br>*Streptomyces fradiae*<br>*Streptomyces cyanogenus*	AGO50627.1<br>BAV17016.1<br>ARO44652.1<br>CAF31368.1<br>AAF72552.1<br>AAD13549.1
**65**	LRR80_00505	Oxidoreductase	960	GcnS8, 89.34 %<br>sqnS8, 83.65%<br>sprS, 86.79%<br>schS1, 76.85%	*Streptomyces lusitanus*<br>*Streptomyces sp.*<br>*Streptomyces sp. TK08046*<br>*Streptomyces sp. SCC 2136*	AGO50628.1<br>ARO44666.1<br>BAV17017.1<br>CAF31369.2
**66**	LRR80_00506	Acetyl-CoA carboxylase, carboxyl transferase	1536	GcnP, 97.85%<br>sqnP, 95.67%<br>sprU, 96.06%<br>Homolog in rubrolone A, 94.09%	*Streptomyces lusitanus*<br>*Streptomyces sp.*<br>*Streptomyces sp. TK08046*<br>*Streptomyces sp. KIB-H033*	AGO50629.1<br>ARO44649.1<br>BAV17019.1<br>AOZ61215.1
**67**	LRR80_00507	Acyl-CoA carboxylase epsilon subunit	192	ChlI, 62.07%	*Streptomyces antibioticus*	AAZ77683.1
**68**	LRR80_00508	FAD linked oxidase protein	1590	GcnQ, 95.09%<br>sqnQ, 86.96%<br>sprY, 84.69%<br>schA26, 72.18%	*Streptomyces lusitanus*<br>*Streptomyces sp.*<br>*Streptomyces sp. TK08046*<br>*Streptomyces sp. SCC 2136*	AGO50630.1<br>ARO44647.1<br>BAV17021.1<br>CAH10126.1

**69**	LRR80_00509	Hypothetical protein	438	sqnEE, 84.03%<br>sprZ, 82.64%	*Streptomyces sp.*<br>*Streptomyces sp. TK08046*	ARO44681.1<br>BAV17022.1
**70**	LRR80_00510	Response regulator	711	GcnR, 95.34%<br>sprR3, 87.66%<br>sqnR, 86.86%	*Streptomyces lusitanus*<br>*Streptomyces sp. TK08046*<br>*Streptomyces sp.*	AGO50631.1<br>BAV17023.1<br>ARO44672.1
**71**	LRR80_00511	Oxidoreductase	354	SCO6269, 78.95%<br>schA18, 76.72%<br>sqnU, 71.55%	*Streptomyces coelicolor A3(2)*<br>*Streptomyces sp. SCC 2136*<br>*Streptomyces sp.*	CAB60188.1<br>CAF31373.1<br>ARO44661.1
**72**	LRR80_00512	Hypothetical protein	246	n.a.		
**73**	LRR80_00513	Integral membrane protein TerC family	954	Orf+1, 91.17%	*Streptomyces lusitanus*	AGO50634.1
**74**	LRR80_00514	Siderophore: IucA_IucC	1623	n.a.		
**75**	LRR80_00515	Siderophore: IucA_IucC	1506	n.a.		
**76**	LRR80_00516	Hypothetical protein	1272	n.a.		
**77**	LRR80_00517	Hypothetical protein	801	n.a.		
**78**	LRR80_00518	Hypothetical protein unknown function	531	arpX, 73.71%	*Streptomyces argillaceus*	SCO70312.1
**79**	LRR80_00519	YciI family protein	549	n.a.		
**80**	LRR80_00520	Transcriptional regulator	627	n.a.		
**81**	LRR80_00521	Aminotransferase	1209	n.a.		

[a] Identity in amino acid between predicted gene product of *Streptomyces* sp. RO-S4 and its nearest homologue.

n.a.: not annotated

```
gnl|LBBM|ROS4_2 [gcode=11] [organism=Streptomyces sp.] [strain=RO-S4]
1989 nucleotides in sequence
Mean G+C content = 72.8%

1.



               g
             t-a
             g-c
             c-g
             g-c
             g-c
             g-c
             t.t
             g-c     aa
            a   gccgc  a
    cct    c    +! !!  g
   g   gccg     tgacg  c
   g   !!!!     g    ct
   c   cggc      g
    ccg          g
            g-cc g
            g-c g
            g-c
            c-g
            g-c
           t   a
           t   c
            ggc


    tRNA-Ala(ggc)
    76 bases, %GC = 80.3
    Sequence c[568,643]
```

Figure S76. $tRNA_{ala}$ predicted by Aragorn in the complementary strand of the LRR80_00492 ORF region.

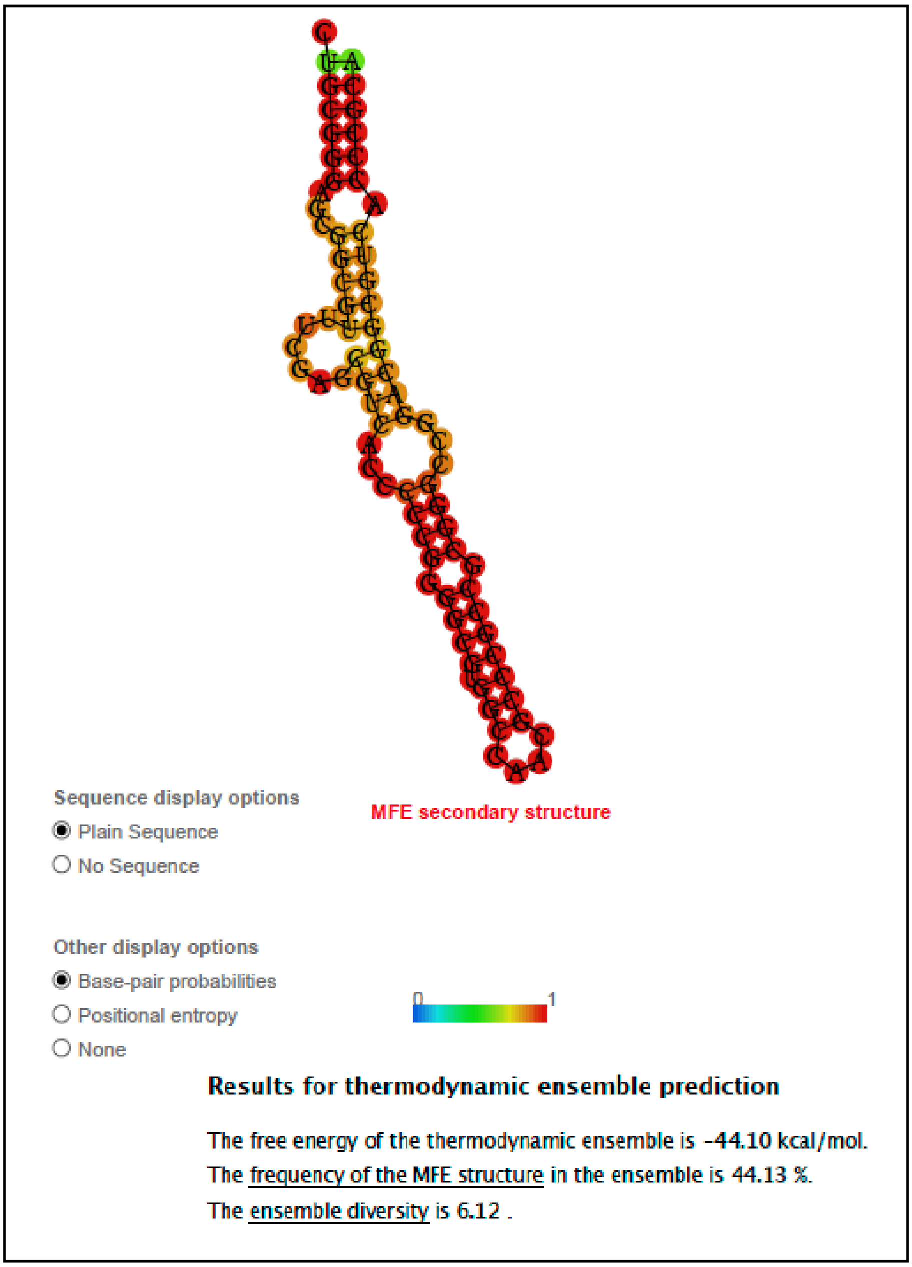

Figure S77. RNA-fold predicted RNA secondary structure of the region predicted by Aragorn to code (in the complementary strand) a tRNAala in the LRR80_00492 ORF region gene.

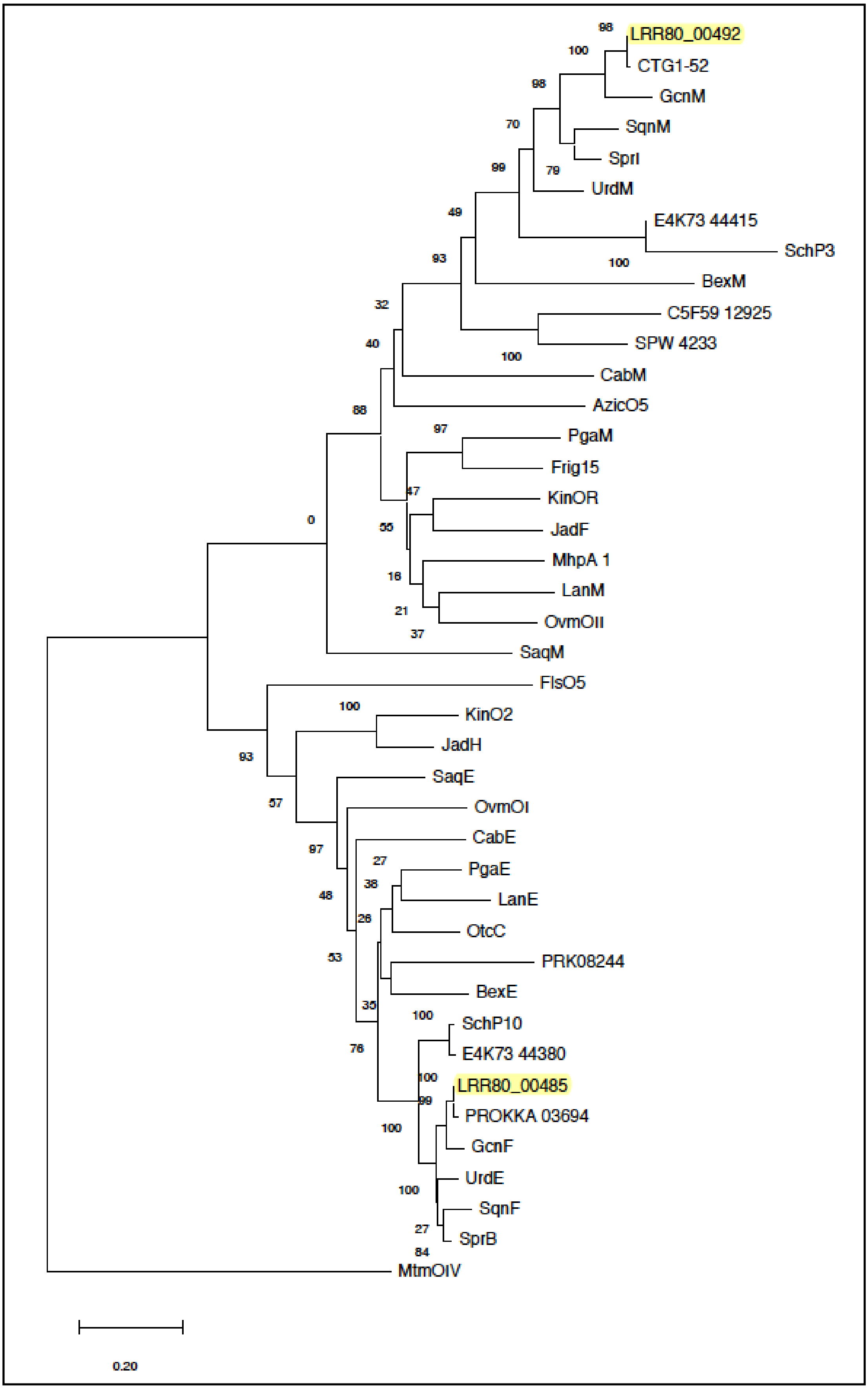
LRR80_00492
CTG1-52
GcnM
SqnM
SprI
UrdM
E4K73 44415
SchP3
BexM
C5F59 12925
SPW 4233
CabM
AzicO5
PgaM
Frig15
KinOR
JadF
MhpA 1
LanM
OvmOII
SaqM
FlsO5
KinO2
JadH
SaqE
OvmOI
CabE
PgaE
LanE
OtcC
PRK08244
BexE
SchP10
E4K73 44380
LRR80_00485
PROKKA 03694
GcnF
UrdE
SqnF
SprB
MtmOIV
0.20

Figure S78. Phylogenetic reconstruction (Neighbor Joining) of FAD dependent oxidases as described in the text. Where:

PcpB Pentachlorophenol 4-monooxygenase [*Sphingobium chlorophenolicum* L-1] AAF15368
RdmE Aklavinone C11-hydroxylase [*Streptomyces purpurascens* ATCC 25489] AAA83424
TcmG Tetracenomycin A2 hydroxylase [*Streptomyces glaucescens*] AAA67511
DnrF Aklavinone C-11 hydroxylase [*Streptomyces peucetius subsp. caesius* ATCC 27952] AAA62496
LRR80_00492 Oxidase-Reductase (This study) [*Streptomyces* sp. S4]
CTG1-52 Oxidase-Reductase (Grincamycins) [*Streptomyces* sp. CZN-748]
GcnM Oxygenase-reductase (Grincamycins) [*Streptomyces lusitanus* SCSIO LR32] AGO50615
UrdM Oxygenase-reductase (Urdamycin) [*Streptomyces fradiae* Tu 2717] AFU51427
SqnM Monooxygenase (Saquayamycin) [*Streptomyces* sp. KY40-1] ARO44645
SprI Oxygenase-reductase (Saprolmycin) [*Streptomyces* sp. TK08046] BAV17004
C5F59_12925 SDR family oxidoreductase (Lugdunomycin) [*Streptomyces* sp. QL37] PPQ57494
E4K73_44415 SDR family oxidoreductase (Baikalomycin) [*Streptomyces* sp. IB201691-2A2] WP_143644098
SPW_4233 monooxygenase FAD-binding (Kiamycin) [*Streptomyces* sp. W007] EHM27510
BexM oxygenase-reductase (BE-7585A) [*Amycolatopsis orientalis subsp. vinearia*] ADI71448
SchP3 oxidase-reductase (Sch 47554) [*Streptomyces* sp. SCC 2136] CAH10112
PgaM two-domain mono-oxygenase/angucyclinone (Gaudimycins) [*Streptomyces* sp. PGA64] AAK57530
CabM monooxygenase FAD-binding (Gaudimycin-B) [*Streptomyces* sp H021] DD106591
MhpA_1 3-(3-hydroxy-phenyl) propionate/3-hydroxycinnamic acid hydroxylase (Fridamycin-A) [*Actinomadura* sp. RB99] MBD2892419
Frig15 oxygenase-reductase (Frigocyclinone) [*Streptomyces griseus* NTK97] QDG00821
SaqM putative oxygenase (Saquayamycin Z) [*Micromonospora* sp. Tu 6368] ACP19358
LanM oxygenase homolog (Landomycin) [*Streptomyces cyanogenus* S136] AAD13541
MtmOIV oxygenase (Mithramycin) [*Streptomyces argillaceus* ATCC 12956] CAK50794
OvmOII oxygenase (Oviedomycin) [*Streptomyces antibioticus* ATCC 11891] CAG14970
AzicO5 oxygenase (Azicemicin) [*Kibdelosporangium* sp. MJ126-NF4] ADB02848
FlsO5 polyketide oxygenase CabE (Fluostatin) [*Micromonospora rosaria* SCSIO N160] ALJ9986
KinOR oxygenase reductase-like protein (Kinamycin) [*Streptomyces murayamaensis*] AAO65351
LRR80_00485 monooxygenase FAD-binding (This study) [*Sreptomyces* sp.S4]
PROKKA_03694 monooxygenase FAD-binding (Grincamycins) [*Streptomyces* sp. CZN-748]
GcnF Oxidoreductase [Streptomyces lusitanus SCSIO LR32] AGO50609
SaqE Putative oxygenase (Saquayamycin Z) [*Micromonospora* sp. Tu 6368] ACP19351
SqnF monooxygenase (Saquayamycin) [*Streptomyces* sp. KY40-1] ARO44651
SprB Putative oxygenase (Saprolmycin) [*Streptomyces* sp. TK08046] BAV16997
SchP10 putative oxygenase (Sch 47554 and Sch 47555) [*Streptomyces* sp. SCC 2136] CAH10119
PRK08244 FAD-dependent monooxygenase (Lugdunomycin) [*Streptomyces* sp. QL37] WP_104785789
E4K73_44380 monooxygenase (Baikalomycins) [*Streptomyces* sp. IB201691-2A2] TRO56975
PgaE prejadomycin C12- and C12b- hydroxylase (Prejadomycin) [*Streptomyces* sp. PGA64] AAK57522
OtcC Anhydrotetracycline monooxygenase (Fridamycin-A) [*Actinomadura* sp. RB99] MBD2892418
LanE oxygenase homolog (Landomycin) [*Streptomyces cyanogenus* S136] AAD13534
MtmOIV oxygenase (Mithramycin) [*Streptomyces argillaceus* ATCC 12956] CAK50794
OvmOI oxygenase (Oviedomycin) [*Streptomyces antibioticus* NRRL 3238] ARK36153
KinO2 oxygenase-like protein (Kinamycin) [*Streptomyces murayamaensis*] AAO65352
BexE putative oxygenase (BE-7585A) [*Amycolatopsis orientalis subsp. vinearia*] ADI71441
CabE monooxygenase FAD-binding (Gaudimycin-B) [*Streptomyces* sp H021] DD106591

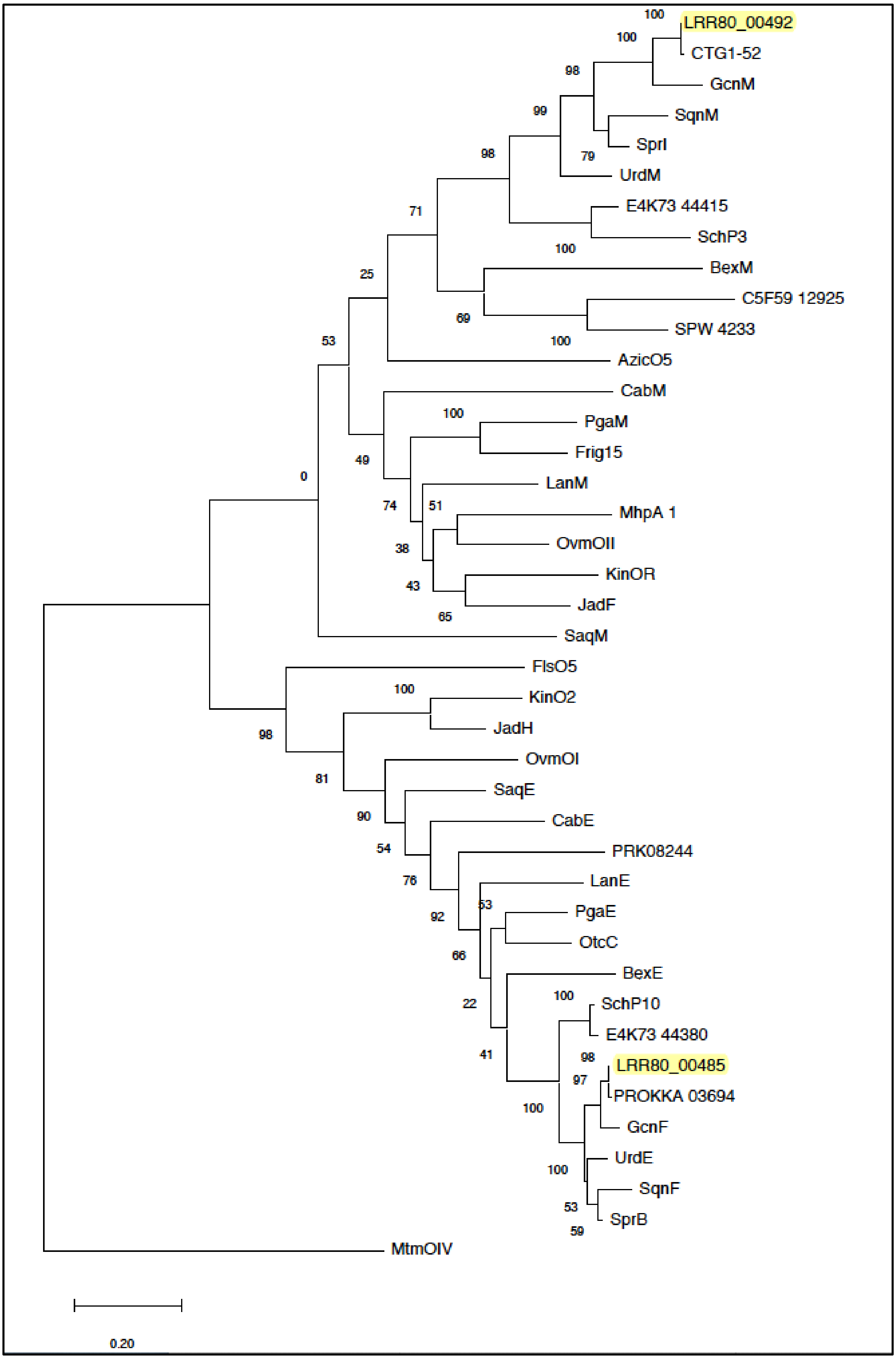

LRR80_00492
CTG1-52
GcnM
SqnM
SprI
UrdM
E4K73 44415
SchP3
BexM
C5F59 12925
SPW 4233
AzicO5
CabM
PgaM
Frig15
LanM
MhpA 1
OvmOII
KinOR
JadF
SaqM
FlsO5
KinO2
JadH
OvmOI
SaqE
CabE
PRK08244
LanE
PgaE
OtcC
BexE
SchP10
E4K73 44380
LRR80_00485
PROKKA 03694
GcnF
UrdE
SqnF
SprB
MtmOIV
0.20

Figure S79. Phylogenetic reconstruction (Maximum Likelihood) of FAD dependent oxidases as described in the text.

PcpB Pentachlorophenol 4-monooxygenase [*Sphingobium chlorophenolicum* L-1] AAF15368
RdmE Aklavinone C11-hydroxylase [*Streptomyces purpurascens* ATCC 25489] AAA83424
TcmG Tetracenomycin A2 hydroxylase [*Streptomyces glaucescens*] AAA67511
DnrF Aklavinone C-11 hydroxylase [*Streptomyces peucetius subsp. caesius* ATCC 27952] AAA62496
LRR80_00492 Oxidase-Reductase (This study) [*Streptomyces* sp. RO-S4]
CTG1-52 Oxidase-Reductase (Grincamycins) [*Streptomyces* sp. CNZ-748]
GcnM Oxygenase-reductase (Grincamycins) [*Streptomyces lusitanus* SCSIO LR32] AGO50615
UrdM Oxygenase-reductase (Urdamycin) [*Streptomyces fradiae* Tu 2717] AFU51427
SqnM Monooxygenase (Saquayamycin) [*Streptomyces* sp. KY40-1] ARO44645
SprI Oxygenase-reductase (Saprolmycin) [*Streptomyces* sp. TK08046] BAV17004
C5F59_12925 SDR family oxidoreductase (Lugdunomycin) [*Streptomyces* sp. QL37] PPQ57494
E4K73_44415 SDR family oxidoreductase (Baikalomycin) [*Streptomyces* sp. IB201691-2A2] WP_143644098
SPW_4233 monooxygenase FAD-binding (Kiamycin) [*Streptomyces* sp. W007] EHM27510
BexM oxygenase-reductase (BE-7585A) [*Amycolatopsis orientalis subsp. vinearia*] ADI71448
SchP3 oxidase-reductase (Sch 47554) [*Streptomyces* sp. SCC 2136] CAH10112
PgaM two-domain mono-oxygenase/angucyclinone (Gaudimycins) [*Streptomyces* sp. PGA64] AAK57530
CabM monooxygenase FAD-binding (Gaudimycin-B) [*Streptomyces* sp H021] DD106591
MhpA_1 3-(3-hydroxy-phenyl) propionate/3-hydroxycinnamic acid hydroxylase (Fridamycin-A) [*Actinomadura* sp. RB99] MBD2892419
Frig15 oxygenase-reductase (Frigocyclinone) [*Streptomyces* griseus NTK97] QDG00821
SaqM putative oxygenase (Saquayamycin Z) [*Micromonospora* sp. Tu 6368] ACP19358
LanM oxygenase homolog (Landomycin) [*Streptomyces cyanogenus* S136] AAD13541
MtmOIV oxygenase (Mithramycin) [*Streptomyces argillaceus* ATCC 12956] CAK50794
OvmOII oxygenase (Oviedomycin) [*Streptomyces antibioticus* ATCC 11891] CAG14970
AzicO5 oxygenase (Azicemicin) [*Kibdelosporangium* sp. MJ126-NF4] ADB02848
FlsO5 polyketide oxygenase CabE (Fluostatin) [*Micromonospora rosaria* SCSIO N160] ALJ9986
KinOR oxygenase reductase-like protein (Kinamycin) [*Streptomyces murayamaensis*] AAO65351
LRR80_00485 monooxygenase FAD-binding (This study) [*Streptomyces* sp. RO-S4]
PROKKA_03694 monooxygenase FAD-binding (Grincamycins) [*Streptomyces* sp. CNZ-748]
GcnF Oxidoreductase [*Streptomyces lusitanus* SCSIO LR32] AGO50609
SaqE Putative oxygenase (Saquayamycin Z) [*Micromonospora* sp. Tu 6368] ACP19351
SqnF monooxygenase (Saquayamycin) [*Streptomyces* sp. KY40-1] ARO44651
SprB Putative oxygenase (Saprolmycin) [*Streptomyces* sp. TK08046] BAV16997
SchP10 putative oxygenase (Sch 47554 and Sch 47555) [*Streptomyces* sp. SCC 2136] CAH10119
PRK08244 FAD-dependent monooxygenase (Lugdunomycin) [*Streptomyces* sp. QL37] WP_104785789
E4K73_44380 monooxygenase (Baikalomycins) [*Streptomyces* sp. IB201691-2A2] TRO56975
PgaE prejadomycin C12- and C12b- hydroxylase (Prejadomycin) [*Streptomyces* sp. PGA64] AAK57522
OtcC Anhydrotetracycline monooxygenase (Fridamycin-A) [*Actinomadura* sp. RB99] MBD2892418
LanE oxygenase homolog (Landomycin) [*Streptomyces cyanogenus* S136] AAD13534
MtmOIV oxygenase (Mithramycin) [*Streptomyces argillaceus* ATCC 12956] CAK50794
OvmOI oxygenase (Oviedomycin) [*Streptomyces antibioticus* NRRL 3238] ARK36153
KinO2 oxygenase-like protein (Kinamycin) [*Streptomyces murayamaensis*] AAO65352
BexE putative oxygenase (BE-7585A) [*Amycolatopsis orientalis subsp. vinearia*] ADI71441
CabE monooxygenase FAD-binding (Gaudimycin-B) [*Streptomyces* sp H021] DD106591
